# Diversity and functional prediction of microbial communities involved in the first aerobic bioreactor of coking wastewater treatment system

**DOI:** 10.1371/journal.pone.0243748

**Published:** 2020-12-10

**Authors:** Jinsi Deng, Baoshan Zhang, Junting Xie, Haizhen Wu, Zemin Li, Guanglei Qiu, Chaohai Wei, Shuang Zhu

**Affiliations:** 1 Guangdong Province Key Laboratory for Biotechnology Drug Candidates, School of Biosciences and Biopharmaceutics, Guangdong Pharmaceutical University, Guangzhou, China; 2 School of Biology and Biological Engineering, South China University of Technology, Guangzhou, China; 3 School of Environment and Energy, South China University of Technology, Guangzhou, China; Tsinghua University, CHINA

## Abstract

The pre-aerobic process of coking wastewater treatment has strong capacity of decarbonization and detoxification, which contribute to the subsequent dinitrogen of non-carbon source/heterotrophic denitrification. The COD removal rate can reach > 90% in the first aerobic bioreactor of the novel O/H/O coking wastewater treatment system during long-term operation. The physico-chemical characteristics of influent and effluent coking wastewater in the first aerobic bioreactor were analyzed to examine how they correlated with bacterial communities. The diversity of the activated sludge microbial community was investigated using a culture-independent molecular approach. The microbial community functional profiling and detailed pathways were predicted from the 16S rRNA gene-sequencing data by the PICRUSt software and the KEGG database. High-throughput MiSeq sequencing results revealed a distinct microbial composition in the activated sludge of the first aerobic bioreactor of the O/H/O system. Proteobacteria, Bacteroidetes, and Chlorobi were the decarbonization and detoxification dominant phyla with the relative abundance of 84.07 ± 5.45, 10.89 ± 6.31, and 2.96 ± 1.12%, respectively. *Thiobacillus*, *Rhodoplanes*, *Lysobacter*, and *Leucobacter* were the potential major genera involved in the crucial functional pathways related to the degradation of phenols, cyanide, benzoate, and naphthalene. These results indicated that the comprehensive understanding of the structure and function diversity of the microbial community in the bioreactor will be conducive to the optimal coking wastewater treatment.

## Introduction

Coking wastewater is generated from the high-temperature carbonization of raw coal, coal gas purification, and refinement of the products generated during coke production. In coking wastewater, the pollutants are typically recalcitrant, highly toxic, and carcinogenic, including inorganic compounds such as ammonia, cyanide (CN^−^) and thiocyanate (SCN^−^), and organic compounds, such as phenolic compounds, polycyclic aromatic hydrocarbons (PAHs), polycyclic nitrogen-containing aromatics, as well as oxygen- and sulfur-containing heterocyclics [1,2]. Coking wastewater is regarded as one of the most toxic industrial effluents, which can cause severely adverse effects on the environment [3]. As the most economical and environmentally friendly treatment process, the activated sludge technique has been widely used to remove pollutants from coking wastewater. Previously, a novel coking wastewater biological treatment process O (first aerobic bioreactor)–H (hydrolytic bioreactor)–O (second aerobic bioreactor) could achieve simultaneous removal of contaminants, especially, the first aerobic bioreactor has a high capacity of decarbonization and detoxification [4]. However, the microbial diversity and function involved in the first aerobic bioreactor of the O/H/O treatment system have not yet been precisely characterized.

Compared to the use of anaerobic activated sludge at the start of the coking wastewater biological treatment process, aerobic activated sludge is more resistant to various toxic pollutants and conducive to significant organic pollutants degradation [5]. Based on our previous research, the first aerobic bioreactor of the O/H/O system can remove more than 90% of the chemical oxidation demand (COD) and the toxic compounds [6]. Aerobic activated sludge can provide a suitable environment for the microorganisms to grow that can improve the efficiency of the removal of pollutants [7,8]. Therefore, the prefixed aerobic process is the key step for high-loading decarbonization, partial degradation of refractory and inhibitory organic compounds to reduce the loading of subsequent processes. However, the bioreactor has not been optimized because of our limited knowledge of diversity and function of the microbial communities involved in this bioreactor.

In view of the powerful pollutant removal capacity of the first aerobic bioreactor, and the significance of the sequent dinitrogen process design, in this study, we tried to illustrate the diversity and function of the microbial communities involved in the first aerobic bioreactor of a full-scale O/H/O coking wastewater treatment system. Pollutant removal efficiency was measured, and the operating parameters were monitored during long-term stable operation. Illumina MiSeq high throughput sequencing was performed to reveal the relative abundance and diversity of the microbial community. Phylogenetic investigation of communities by reconstruction of unobserved states (PICRUSt) was used to predict the function based on 16S rRNA gene information. The aims of this work were to illustrate the diversity of the microbial communities, identify the dominant members of the bacterial community responsible for the removal of major pollutants, and predict the function of the dominant bacterial taxa. Our results provided a scientific basis for the development of new control strategies on nitrogen removal during coking wastewater treatment.

## Materials and methods

### The first aerobic bioreactor of the O/H/O system and sample collection

Coking wastewater activated samples were collected from the plant with permission of the administrator in the Tianjin Iron and Steel Corporation, Shexian Hebei Province of China. This article does not contain any studies with human, animals or endangered or protected species.

The first aerobic bioreactor of a full-scale three-phase fluidized bed biological O/H/O coking wastewater treatment system in the Tianjin Iron and Steel Corporation, Shexian Hebei Province of China, was studied. To relieve the accidental event shock, this system contains two separate, independent parallel subsystems, named south and north subsystem, respectively. This O/H/O coking wastewater treatment system has been in operation for 7 years and can maintain a stable performance during operation. This system has the ability to process about 2,400 ± 160 m^3^ daily.

Activated sludge samples were collected in triplicate on different days from the corresponding south and north subsystems of the first aerobic bioreactor in May 2018. All samples were placed in ice boxes during the sampling and transportation to the laboratory. Aliquots (2–3 mL) of each sample were centrifuged at 12,000 g for 10 min at 4°C. The cell pellets were washed twice with sodium phosphate buffer (120 mM, pH 8.0) and stored at −20°C before DNA was extracted.

### Physico-chemical analysis

The collected coking wastewater samples were centrifuged at 3,500 g for 3 min. Subsequently, the supernatants were used for analysis of COD, biochemical oxygen demand (BOD), total suspended solids (TSS) by the standard spectrophotometric methods (APHA 2005). Mixed liquor suspended solids (MLSS) was measured according to standard methods (APHA 2006). The contents of ammonium, phenolic compounds, sulfides (S^2−^), total phosphorus (TP), and total nitrogen (TN) were analyzed by the colorimetric methods with a spectrophotometer (Genesys TM-5, Spectronic Inc., USA) [9]. The concentration of CN^−^ was measured by the pyridine-pyrazolone method after distillation [9]. SCN^−^ was measured using a UV-vis spectrophotometer (UV-1800, Shimadzu, Japan) by the colorimetric method with ferric nitrate [10]. The total organic carbon (TOC) was measured by TOC analyzer (Shimadzu TOC-VCPH, Japan). The dissolved oxygen (DO) concentration was measured using a DO meter (CellOX 3310i, WTW, Germany), and the pH was monitored with a pH meter (PHS-3D, Shanghai Precision & Scientific Instrument Co. Ltd., China). Concentrations of PAHs were determined using the Agilent 7890A GC equipped with 5975C mass selective detector with a 30 m × 0.25 mm i.d. × 0.25 μm film fused silica capillary column (HP-5 MS, Agilent Technology) under the selected ion mode.

Reaction efficiency and microbial population were directly affected by the design of bioreactor. In a previous study, an internal-loop multiphase airlift fluidized-bed reactor has been developed to deal with high COD load rates (> 2.00 kg COD/m^3^d) [11], which was applied in this study. The internal-loop design ensured efficient mixing and mass transfer with low energy consumption [12]. The average pH of the first aerobic bioreactor was kept at 7.35 ± 0.11. Long-term operating temperatures were relatively constant between 21 to 25°C (**Table 1**) and the DO level was maintained in the range of 2.95–3.57 mg/L. Sludge retention time (SRT) was 8 ± 2 days. The hydraulic retention time (HRT) was 60 ± 8 h, and the concentrations of activated sludge ranged from 3,700 to 4,700 mg/L. The operational parameters of the first aerobic bioreactor are listed in **Table 1**.

**Table 1:** Operational parameters of the first aerobic bioreactor.

T(°C)	pH	DO (mg/L)	HRT(h)	SRT(d)	MLSS (mg/L)	Volume (m^3^)	COD load rate (kg COD/m^3^d)	COD removal rate (kg COD/m^3^d)
23(21–25)	7.35 ± 0.11	3.26 ± 0.31	60 ± 8	8 ± 2	4200 ± 500	3600	> 2.00	1.60

T: Temperature; DO: Dissolved oxygen; HRT: Hydraulic retention time; SRT: Sludge retention time; MLSS: Mixed liquor suspended solids.

### MiSeq sequencing of 16S rRNA genes

Microbial DNA was extracted using the PowerSoil^TM^ DNA isolation kit (Mobio, USA) according to the manufacturer’s instructions. The DNA quality was assessed by the ratios of 260/280 nm and 260/230 nm absorption measured by the ND-2000 spectrophotometer (Thermo Fisher Scientific, USA), and agarose gel electrophoresis. The V4 hypervariable region of the 16S rRNA gene was PCR-amplified (triplicate reactions for each sample) using primers F515 (5’-GTGCCAGCMGCCGCGGTAA-3’) and R806 (5’-GGACTACVSGGGTATCTAAT-3’), following the procedures described by Zhu et al. (2016) [13]. These primers are universal for almost all bacterial and archaeal taxa [14,15]. The obtained sequences were deposited in the NCBI Sequence Read Archive under the accession number PRJNA615645.

### Sequence analysis

The composition of the PCR products of the V4 region of 16S rRNA genes was determined by Illumina MiSeq PE300 sequencing platform. Samples were individually barcoded to enable multiplex sequencing. After MiSeq sequencing, the raw data was processed and analyzed following the pipelines of Mothur (v1.35.1) [16] and QIIME v 1.7 (quantitative insights into microbial ecology) [17]. Operational taxonomic units (OTUs, defined at the 97% sequence similarity level) were picked using the average neighbor method after Needleman alignment and a single-linkage pre-cluster procedure. Taxonomic classification was performed using the RDP Classifier at 80% confidence threshold, by default. Alpha-diversity (number of OTUs, Chao 1, and Simpson and Shannon indices) was statistically analyzed via rarefaction, with all data sets normalized to 17,668 reads per sample. Good’s coverage estimators representing subsample coverage were calculated using the Mothur software.

The functional microbiota content was predicted based on the 16S rRNA gene sequencing data using the PICRUSt (v 1.0.0.6) software [18]. This analysis was derived from the observation of an association between phylogeny and gene content. For the analysis, OTUs were closed-reference picked against the GreenGenes (released 13.5) database using QIIME v 1.7 according to the online protocol [19], removing the OTUs not matching the GreenGenes 13−5 reference sequences at 97% similarity. The OTUs were normalized by dividing their relative abundance values by known or predicted 16S rRNA gene copy numbers prior to final metagenomic predictions. The resulting data set was rarefied at 17,668 16S rRNA sequences per sample. Predicted functional counts were rarefied to the same depth, and relative abundance analyses were calculated by comparing Kyoto Encyclopedia of Genes and Genomes (KEGG, http://www.genome.jp/kegg/) orthology values (levels 1, 2, and 3) in activated sludge samples from the first aerobic bioreactor. The potential metabolic functions for pollutant removal were established by the comparison of all sequences from the coking wastewater activated sludge to the KEGG using functional assignments from PICRUSt. The accuracy of metagenome predictions was measured by the Nearest Sequenced Taxon Index (NSTI), whose lower values indicate a closer mean relationship [18]. For each OTU in a sample, the sum of the branch lengths between that OTU in the GreenGenes tree to the closest tip in the sequenced genomic tree was weighted by the relative abundance of the OTU. Subsequently, all OTU scores were added up to obtain a single NSTI value for each microbiome sample. The NSTI values for each sample were calculated by PICRUSt, and NSTI scores and PICRUSt accuracies for all metagenome validation datasets were compared.

### Data analysis

The statistical significances of the differences in the concentrations of physico-chemical pollutants, the microbial diversity indices, and the abundances of phyla, genera, OTUs, and gene functions were analyzed by independent-samples student *t* tests with the SPSS Statistics Software version 25 (IBM), considering statistically significant differences those with *p* value < 0.05. The stacked bar plots and interleaved bar plots were constructed by GraphPad Prism (v 8.0.2). The heat map was constructed based on OTUs with relative abundance > 0.5% in at least one sample using the pheatmap packages of R software (v 3.6.1). Redundancy analysis (RDA) based on genera and pollutants was conducted using the vegan package and the ggplot2 package of R software (v 3.6.2).

## Results and discussion

### Physico-chemical characteristics of the first aerobic bioreactor

The characteristics of the coking wastewater were defined and quantified with 13 coking wastewater indices, including COD, BOD, phenols, CN^−^, SCN^−^, ammonia, TN, TP, TSS, TOC, sulfides, oil, and PAHs. Physico-chemical characteristics of influent and effluent in the first aerobic bioreactor were measured as the efficiency indicator (**Table 2**). Based on the results of the independent-samples *t* tests, the removal efficiencies for all contaminants did not significantly differ between the south and the north subsystems (*p >* 0.05). The influent and effluent COD concentrations in the bioreactor were 5,167 ± 270 mg/L and 492 ± 50 mg/L, respectively. It was observed that the COD removal rate could be maintained at above 90% with high organic loading in the bioreactor. The influent BOD was 1,993 ± 185 mg/L, and its removal rate reached 97.19 ± 0.38%. It should be noted that the BOD/COD ratio of the influent was 0.38 ± 0.02, and the corresponding value in the effluent was 0.11 ± 0.01, indicating most of the compounds were degraded. Phenolics, including phenol, o-, m-, and p-cresol, are the main organic constituents of coking wastewaters and account for most of the total COD [20]. The phenols removal efficiency reached 99.59%_._ SCN^−^ is a CN^−^ derivative with lower toxicity [21], and SCN^−^ removal efficiency reached 92.49 ± 0.50%. Compared with the removal of phenol and CN^−^, SCN^−^ degradation is the slowest and most sensitive process. Therefore, it determines the HRT required for the treatment of coking wastewater [22]. The concentration of CN^−^ decreased from 39.0 ± 7.0 mg/L to 0.9 ± 0.6 mg/L, and the removal rate was up to 97.95 ± 1.21%. In previous studies, CN^−^ not only significantly inhibited degradation of SCN^−^ [22], but was also one of the most toxic chemicals for living organisms [23,24].

**Table 2:** Physico-chemical characteristics of influent and effluent in the first aerobic bioreactor of the north and the south subsystem.

Unit	Influent		Effluent	
	N	S	N	S
COD	5135 ± 238	5209 ± 227	485 ± 43	502 ± 39
BOD	2015 ± 162	1988 ± 180	55 ± 10	58 ± 11
Phenols	987 ± 78	1010 ± 79	4.02 ± 0.9	4.26 ± 0.7
Cyanides	38± 6	41 ± 5	0.8 ± 0.3	0.9 ± 0.6
Thiocyanate	550 ± 65	581 ± 68	40 ± 5	44 ± 8
TN	462 ± 42	489 ± 48	423 ± 35	444 ± 29
NH_4_^+^-N	98 ± 9	97 ± 7	78 ± 9	75 ± 6
TP	3.08 ± 0.25	2.06 ± 0.45	0.32 ± 0.10	0.29 ± 0.09
TSS	300 ± 40	283 ± 34	41 ± 8	38 ± 9
TOC	995 ± 47	1002 ± 46	699 ± 40	742 ± 44
Sulfides	148 ± 8	151 ± 6	14.8 ± 2.2	8.7 ± 0.9
Oils	43 ± 21	46 ± 21	46 ± 5	45 ± 8
PAHs	291 ± 43	316 ± 33	134 ±19	150 ± 22

COD: Chemical oxygen demand; TN: Total nitrogen; N: First aerobic bioreactor of the north subsystem; S: First aerobic bioreactor of the south subsystem; All concentrations are in mg/L.

The removal efficiency for PAHs, a typical group of toxic pollutants, was 52.74 ± 2.03%. In addition, sulfide concentration decreased from 149.0 ± 9.0 mg/L to 12.4 ± 4.6 mg/L after the bioreactor, and the removal rate reached 91.9 ± 2.8%. The pH values ranged between 7.24 and 7.46. On the contrary, previous study showed that the prefix anaerobic bioreactor of traditional treatment processes had limited pollutant removal efficiency. One such example is the A/A/O treatment process that had low pollutant removal efficiency and the effluent quality did not meet the wastewater discharge standard in China [25]. Some literatures also reported below 35.00% COD removal by mesophilic anaerobic treatment [13,26,27]. In A/O/O system, the CN^−^ removal rate was below 64.71% [13,28]. These results confirmed that the prefix aerobic bioreactor can remove > 90% of the COD, including toxic compounds of SCN^−^ and CN^−^, with a higher efficiency than the prefix anaerobic bioreactor of traditional treatment processes, such as A/O, A/A/O, A/O/H/O, A/O/O, and SBR [13,27–29]. Our results showed that the first aerobic bioreactor could efficiently decarbonize and detoxify coking wastewater, as well as majority of toxic pollutants including phenol, CN^−^, SCN^−^, and PAHs, thereby facilitating microbial growth and biodegradation in the subsequent bioreactors.

### Phylogenetic diversity analysis of sequences

MiSeq sequencing was used to analyze the bacterial and archaeal 16S rRNA genes across the six samples from the coking wastewater activated sludge of the first aerobic bioreactor. In total, 72,034 and 74,132 effective sequences were retrieved from the north and the south subsystem, respectively. The effective sequences and OTUs of each sample are presented in **Table 3**. We assigned these sequences into different OTUs, using RDP Classifier with 3% of nucleotide cutoff. The OTUs of activated sludge samples from N1, N2, N3, S1, S2, and S3 were identified based on a subset of 17,668 randomly selected sequences, with 539, 471, 543, 464, 510, and 530 OTUs, respectively. The effective sequences and OTUs were not significantly different between the north and the south subsystems of the first aerobic bioreactor (*t* test, *p =* 0.300 and 0.300). Results of good’s coverage in each sample ranged from 99.06 to 99.25%, indicating that sampling depth was sufficient to accurately characterize the bacterial community (**Table 3**). The rarefaction curves of OTUs from six activated sludge samples were relatively flat, demonstrating that the sequencing data were reasonable (**S1 Fig**). Shannon-Weaver index (H) (3.07–3.64) and Simpson (1-D) (0.58–0.71) richness were calculated to estimate the internal (within sample) complexity of individual microorganic populations (**Table 3**). Based on these results, the microbial diversity in the activated sludge was moderate, although more complex than that in some extreme environments, such as hydrothermal springs [30] and acid mine drainage [31], but simpler than that of activated sludge from WWTPs [32,33] and soil [34]. Compared with the first anaerobic bioreactor of the traditional coking wastewater treatment A/O/O system, the bioreactor evaluated here had higher values of the Shannon-Weaver index (H), Simpson, and Chao1 [13]. The microbial community in the bioreactor should acclimatize to the operating conditions. Because the first treatment process can switch from anaerobic to aerobic conditions with different DO concentrations, the microbial community richness and diversity in the sludge samples changed dramatically, and the microbial function would make the adaptive to the environment [35].

**Table 3:** Number of quality sequences, OTUs at 0.03 cut-off, richness estimates, and diversity indices of the microbial communities involved in the first aerobic bioreactor of the north and the south subsystem.

Sample ID	Number of quality reads	Number of OTUs	Good’s coverage (%)	Chao 1	Shannon (H')	Simpson (1-D)
N1	26630	539	99.16	820.28	3.46	0.66
N2	18464	471	99.07	628.99	3.31	0.62
N3	26940	543	99.21	775.98	3.64	0.71
S1	19808	464	99.06	639.56	3.46	0.69
S2	26946	510	99.25	728.48	3.29	0.67
S3	27378	530	99.25	762.65	3.07	0.58

The number of OTUs, richness estimates (Chao 1), and diversity indices (Shannon and Simpson) were calculated based on a subset of 17668 sequences randomly sampled for each community. Coverage was calculated according to the Good’s formula. OTU: Operational taxonomic units. Refer to Table 2 for sample abbreviations.

### Taxonomic complexity of the microbial community

**Fig 1:**
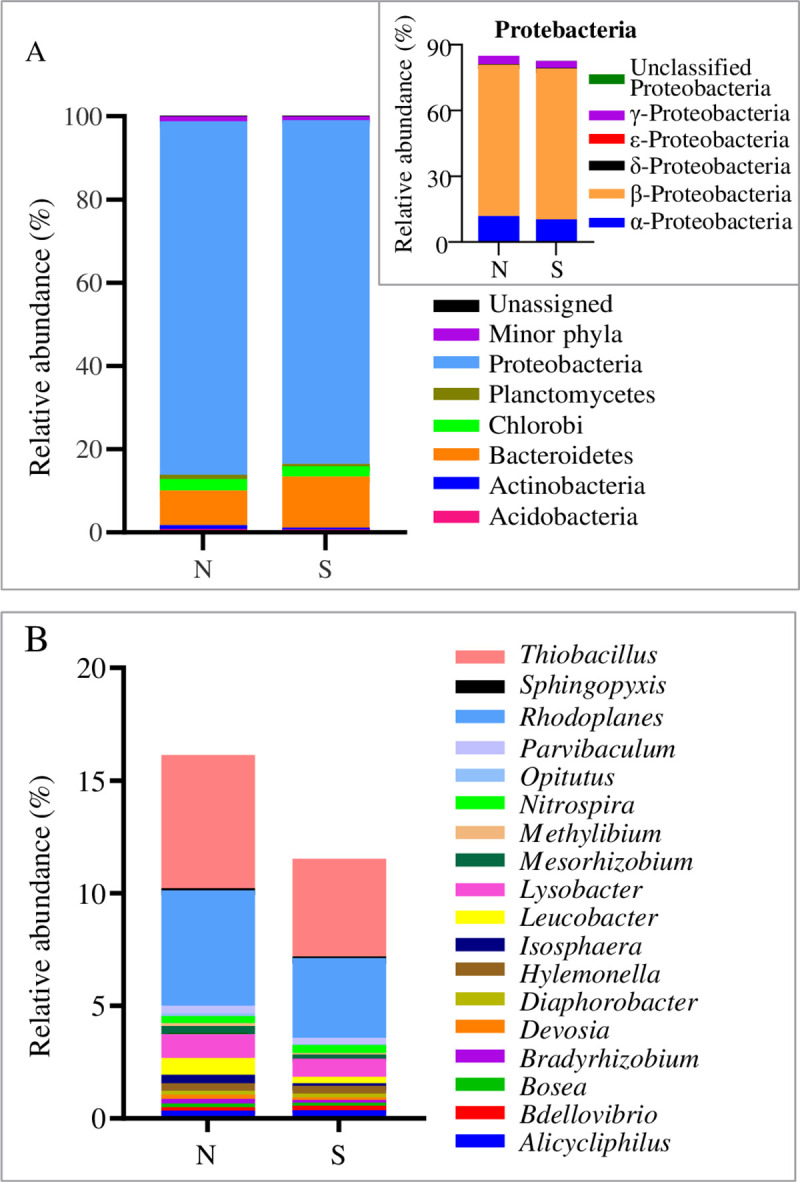
Relative abundance of the phyla/genera in the first aerobic bioreactor. The average values of the three samples were calculated to represent the value of the corresponding sample. Refer to Table 1 for N and S. (A) Phyla (B) Genus.

The effective sequences were assigned into different phylogenetic taxa by RDP Classifier. All classifiable MiSeq sequencing reads across the samples in the north and the south subsystems were assigned to the domain of bacteria. To reveal the microbial community structure and function, the classified sequences were analyzed at phylum, order, family, genus, and OTU levels. There were 23 bacterial phyla in the activated sludge samples taken from the bioreactor, including five classes in the Proteobacteria (**S1 Table**). Phyla with relative abundance > 0.5% in at least one samples were defined as major phyla, and phyla that accounted for a proportion of < 0.5% were defined as minor phyla (**Fig 1A**). The classified phylum results showed that the major phyla were represented by Proteobacteria, Bacteroidetes, and Chlorobi, without significant difference between the two subsystems (*p* > 0.05). The β-Proteobacteria related sequences were the predominant class, with 64.49 to 75.98% relative abundance. According to previous studies, β-Proteobacteria is mainly responsible for organic matter and nutrient removal [36]. The genera *Thauera* and *Thiobacillus*, belonging to β-Proteobacteria can break down aromatic compounds and biotransform SCN^−^, respectively [37,38]. The relative abundance of α-Proteobacteria and γ-Proteobacteria classes were 11.04 **±** 1.32% and 3.49 ± 0.63%, respectively, which played important roles in carbon mineralization in coking wastewater [7,39]. The subdominant phyla were Bacteroidetes (10.89 **±** 6.31%) and Chlorobi (2.96 ± 1.12%). Members of Bacteroidetes can degrade residual recalcitrant substances and high-molecular-weight organic compounds [40,41].

To deepen our understanding of the microbial community composition, the 23 identified phyla were further analyzed, and 79 genera were identified (**S3 Table**). Relative abundance of genus > 0.1% in at least one sample was defined as major genus. Taxa that account for a proportion of < 0.1% were defined as minor genus (**Fig 1B)**. It is possible that these rare taxa may gain predominance during environmental perturbations or toxic shock events and thus are important for the stable performance of coking wastewater treatment systems [13]. The most prevalent genus was *Thiobacillus*, with relative abundance of 5.44 ± 1.28%, which is known for its sulfur metabolism and related to the biodegradation of SCN^−^ in coking wastewater treatment systems [42]. In addition, in another study, SCN^−^ was aerobically degraded to a combination of ammonia, carbonate, and sulfate by several species of the genus *Thiobacillus*, using SCN^−^ as a carbon and nitrogen source [10]. The occurrence and role of *Rhodoplanes* have not frequently been reported in coking wastewater treatment processes [43], but it was a subdominant genus with relative abundance of 4.28 ± 1.08% in this study. Some strains of this genus in municipal wastewater treatment plants can grow on organic carbon sources, such as acetate and pyruvate [44]. The genus *Leucobacter* ranked third, with relative abundance values of 0.58 ± 0.41%. Previous metagenomics and RT-qPCR studies have indicated that the *Leucobacter* strain GP is responsible for the initial ipso-hydroxylation of the parent molecule through a sulfonamide monooxygenase encoded by a gene homolog [45]. *Nitrospira* were the major nitrite-oxidizing bacteria (NOB), with relative abundance of 0.34 ± 0.04%, involved in nitrogen removal and carbon consumption [46–48]. The relative abundance of NOB is typically below 1% in activated sludge from coking wastewater treatment plants [27,43,49]. Unclassified genera belonging to the order Burkholderiales represented the predominant sequences, accounting for 62.97 ± 6.40% at the genus level (**S3 Table**). At this level, nearly three quarters of the sequences could not be assigned, suggesting the existence of numerous novel genera in the first aerobic bioreactor of the O/H/O coking wastewater treatment system. Most abundant genera showed no significant difference between the two subsystems (*p* > 0.05). The minor differences may be attributed to the elasticity of the biological diversity, but will not affect their function [50].

Examination of the identified 117 orders in the first aerobic bioreactor further captured the structure of microbial communities at the order level (**S2A Fig and S2 Table**). The predominant order was Burkholderiales, with relative abundance of 64.53 ± 6.53%. This order plays a key role in the removal of aromatic compounds, such as phenol, benzoate, toluene, naphthalene, and phenanthrene [51–54]. The subdominant order Flavobacteriales occupied 9.84 ± 5.74% relative abundance, while that of the order Rhizobiales was 6.38 ± 1.81%. Most aromatic pollutants, including benzene, toluene, ethylbenzene, xylene, and xenobiotic related compounds could be degraded mainly by Rhizobiales, Burkholderiales, Actinomycetales, as reported in a previous study [7].

**Fig 2:**
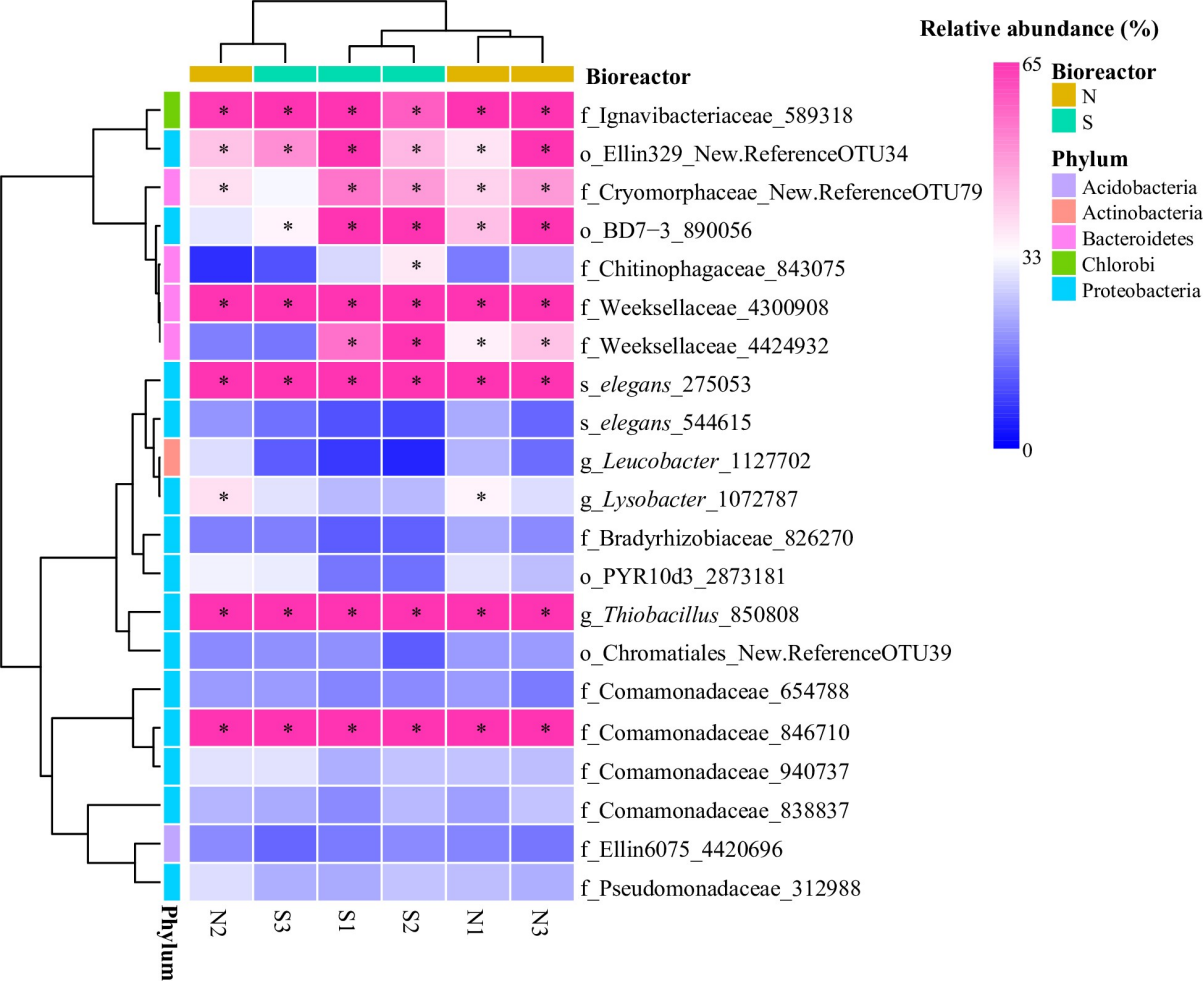
Heatmap of the top 21 OTUs in the first aerobic bioreactor. Color intensity represented the relative abundance of OTUs, with the darker roseate the higher the relative abundance and the darker royal blue the lower the relative abundance. The OTUs of relative abundance > 1% are marked by the star key. Refer to Table 1 for N and S.

We determined 192 families in the first aerobic bioreactor (**S2B Fig and S2 Table**). The distribution of the 21 major OTUs (with relative abundance > 0.5% in at least one sample) is shown in **Fig 2** and **S4 Table**. Comamonadaceae was the predominant bacteria at level of family and OTU, with relative abundance of both more than 60.00%. Four major OTUs, including OTU_846710, OTU_940737, OTU_838837, and OTU_654788, were classified to this family. Our previous study showed that Comamonadeceae played an important role in the biodegradation of phenols, SCN^−^, CN^−^, PAHs, and heterocyclic aromatics in coking wastewater [42]. In addition, the family Comamonadeceae is characterized for the endurance of the toxic compounds of SCN^−^, CN^−^, phenols, PAHs, etc. [55]. Members of Comamonadeceae can also promote PAHs degradation in other organisms, such as species of the strain *Comamonas testosteroni*, which could grow on phenol and its derivatives as carbon and energy sources [56,57]. Two major OTUs (OTU_4300908 and OTU_4424932) belonged to the family Weeksellaceae, which was the subdominant family with 8.43 ± 5.54%. As an aerobic chemoorganotrophic pathogenic family, Weeksellaceae may pose health risks for workers [58]. Nitrosomonadaceae were the dominant ammonium oxidizing bacteria (AOB) with 0.18% relative abundance in the bioreactor of coking wastewater treatment [43,59]. Relative abundance of family Ignavibacteriaceae was more than 2.59%. Interestingly, the *Ignavibacterium* of Ignavibacteriaceae played an important role in the nitrogen removal as a newly identified AOB, which increased the abundance and activities of AOB [60,61]. The long SRT can be conducive to the reproduction and evolution of specific functional microbial populations, such as anammox bacteria, AOB, and NOB [24,62,63]. In a previous study, family Cryomorphaceae played an indispensable role in the carbon and energy cycles of the marine environment [64]. Cryomorphaceae may also play an important role in COD removal in coking wastewater, although this remains to be proved.

### Predictive functional profiling

Function prediction was done to investigate the presence of function pathways related to the biological treatment performance in the first aerobic bioreactor. The north and south subsystems showed satisfactory NSTI values of 0.110 ± 0.002 and 0.114 ± 0.006, respectively. These NSTI of the activated sludge were lower than those recorded for diverse communities such as soil inhabitants 0.170 ± 0.020 [18]. Therefore, the analyzed activated sludge samples from the first aerobic bioreactor provided a data set suitable for examination of PICRUSt predictions.

**Fig 3:**
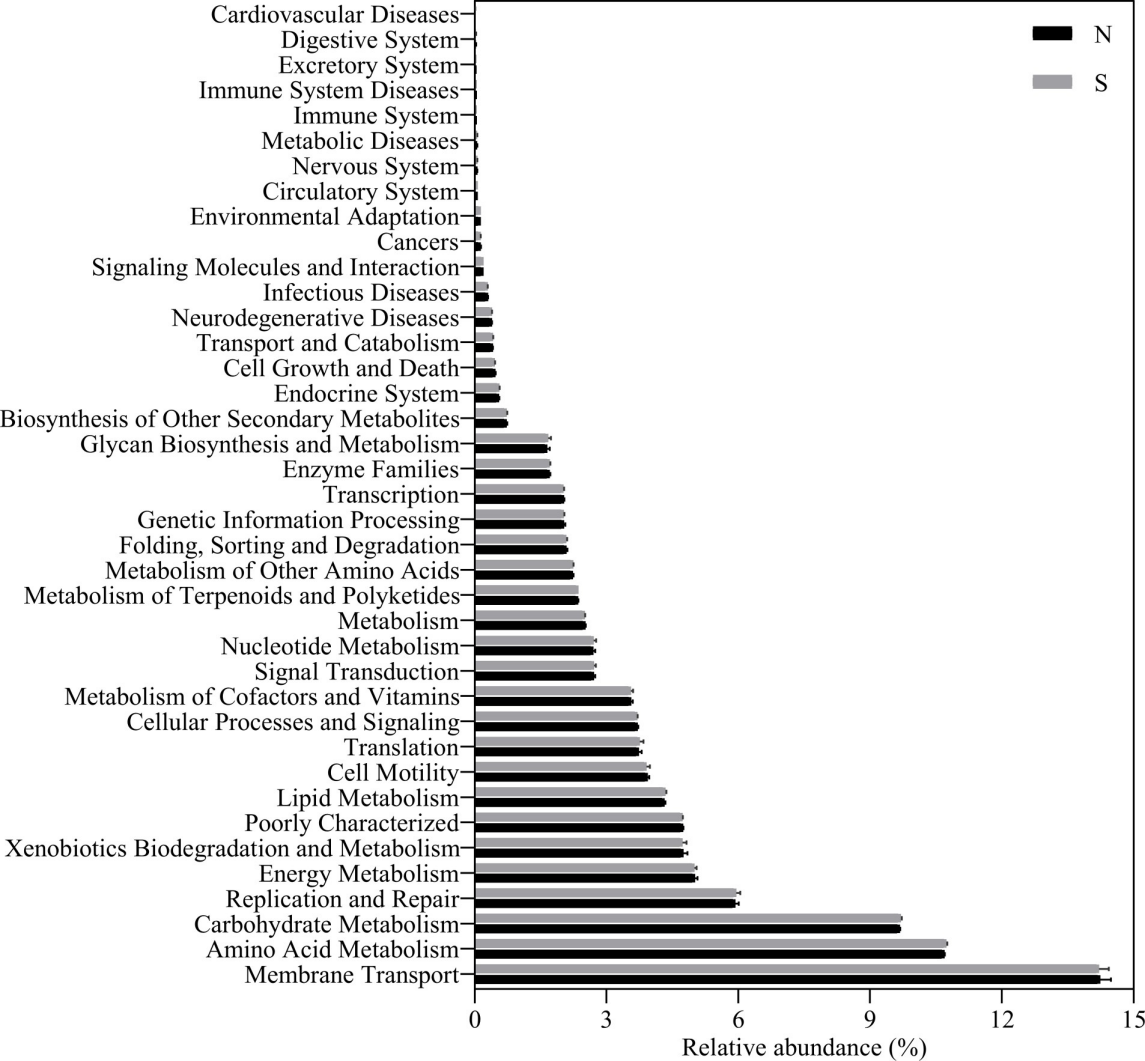
Predicted second-level pathways in KEGG of the bacterial communities in the first aerobic bioreactor. Refer to Table 1 for N and S.

**Fig 4:**
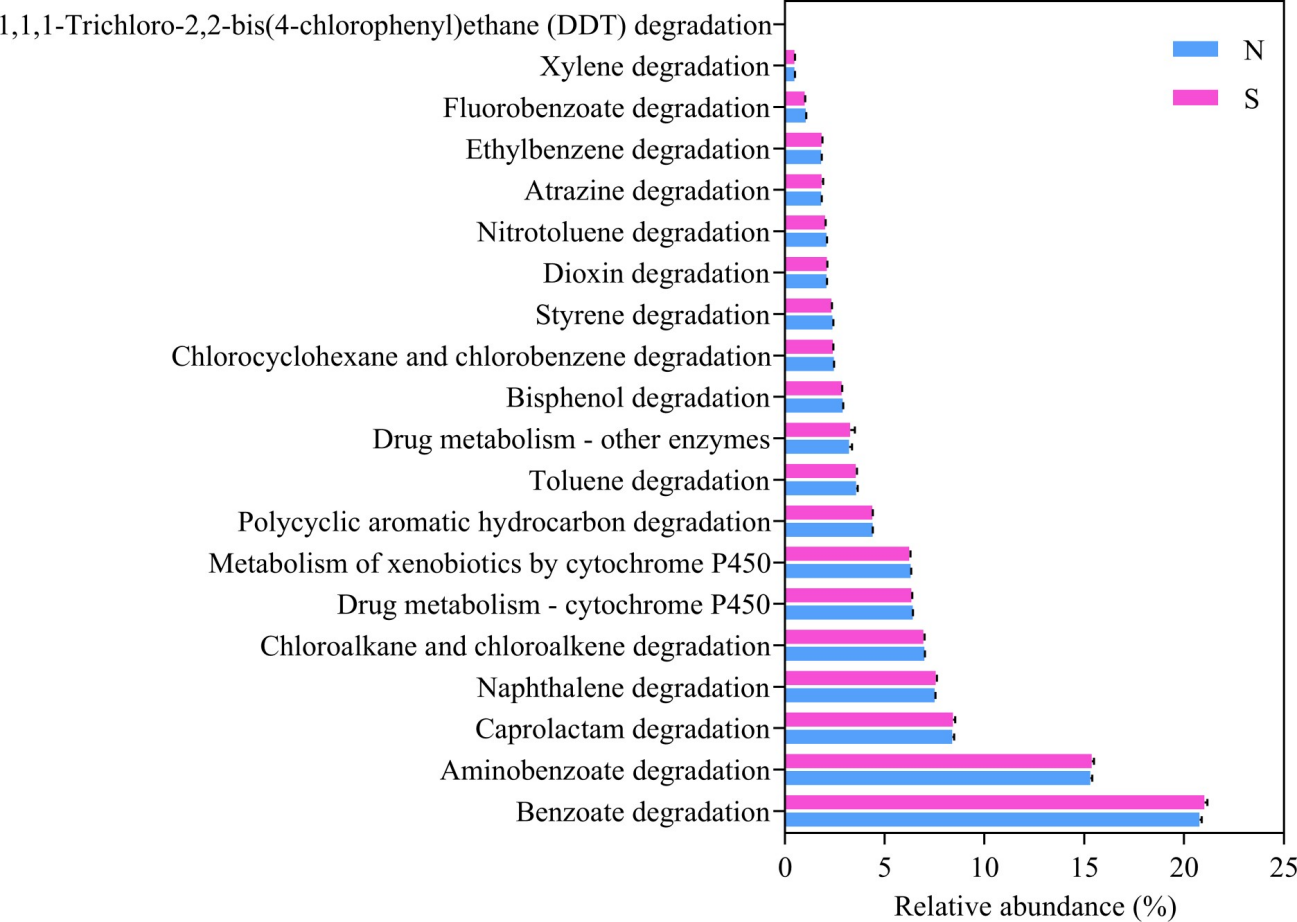
Predicted xenobiotic degradation pathways in KEGG of the first aerobic bioreactor. Refer to Table 1 for N and S.

KEGG pathway database was used to analyze the biodegradation pathways of contaminants. The complex biological behaviour and function of genes can also be intensively and accurately investigated [65]. There were overall 39 of 43 second-level KEGG Orthology categories (KOs) in the predicted metagenomes (**Fig 3**). Predicted ammonia oxidation gene (*amoA*) just possessed the relative abundance of 0.0002% in nitrogen metabolism pathway. Moreover, xenobiotics biodegradation and metabolism pathway had a relatively high relative abundance of 4.74 ± 0.09%, and were involved in the removal of organic exogenous contaminants from coking wastewater. Therefore we further focused on the xenobiotics biodegradation and metabolism pathway. There was relative abundance of the 20 specific metabolic sub-pathways involved in xenobiotics degradation and metabolism pathway (**Fig 4**).

Considering the massive KOs number of xenobiotics degradation and metabolism pathways in KEGG, the top 10 KOs were picked for the analysis, including K01692, K00799, K00257, K00626, K00128, K00100, K01951, K01426, K01061, and K00121, which related to major xenobiotics degradation and metabolism pathways, such as benzoate, aminobenzoate, caprolactam, naphthalene and styrene degradation pathway. The predominant sub-pathway of benzoate degradation (20.92 ± 0.25%) was known to play a role in the degradation of exogenous phenolic compounds, which contribute to the majority of COD in coking wastewater (**Fig 4**). The predictive results of this pathway showed that at least 21 enzymes participated in benzoate degradation (**S3 Fig**). Benzoate degradation is known to play a role in the degradation of a variety of aromatic compounds [66]. The aminobenzoate degradation pathway was an important xenobiotics biodegradation and metabolism pathway that was related to the degradation of phenol and phenyl cyanide (**S4 Fig**). Phenyl cyanide belongs to a type of cyanides that contributes to the high toxicity of coking wastewater. Relative abundance of caprolactam degradation pathway ranked third, and four enzymes were predicted in this pathway related to decarbonization (**S5 Fig**). Naphthalene degradation pathway is related to PAHs, such as naphthalene and its derivatives (1-methylnaphthalene and 2-methylnaphthalene, 1-hydroxymethylnaphthalene, and 2-naphthalenemethanol) (**S6 Fig**). Naphthalene 1,2-dioxygenase (E 1.14.12.12) of this pathway, possessing a wide substrate specificity, permits the cis-hydroxilation of a variety of aromatic compounds including HMW-PAHs [67]. Two major KOs contributed to the styrene degradation pathway, which can degrade the toxic pollutant CN^−^, such as in benzyl cyanide and vinyl cyanide (**S7 Fig**). In the bioreactor, predicted pathways of benzoate degradation, caprolactam degradation, and naphthalene degradation were essential to carbon degradation, such as phenolic compound. Predicted pathways of aminobenzoate degradation and styrene degradation were essential to toxic substance degradation, such as cyanide. The above results indicated that the first aerobic bioreactor could provide function pathways involved in COD removal and detoxification.

### Relationships between major genera and enzymes

Based on literature research, several microbial genera, including *Thiobacillus*, *Comamonas*, *Burkholderiales*, *Pseudomonas*, *Ottowia*, and *Corynebacterium*, are related to the degradation of typical pollutants in coking wastewater, such as phenol, SCN^−^, NHCs, and PAHs, while the biodegradation pathway has not been clearly described [68]. In this study, we attempted to illustrate the relationships of major taxa and enzyme-coding genes in the first aerobic bioreactor of the O/H/O coking wastewater treatment system. Our predicted results are presented in **S5 Table**. The major genus, *Rhodoplanes*, *Lysobacter*, and *Leucobacter*, can provide the five enzymes aldehyde dehydrogenase (E 1.2.1.3), acetyl-CoA C-acetyltransferase (E 2.3.1.9), amidase (E 3.5.1.4), alcohol dehydrogenase (E 1.1.1.1) and carboxymethylenebutenolidase (E 3.1.1.45). It is also predicted that enoyl-CoA hydratase (E 4.2.1.17) was provided by *Rhodoplanes* and *Lysobacter*. These functional prediction results showed that the enzymes were mainly involved in the aminobenzoate, benzoate, styrene, naphthalene, fluorobenzoate, and toluene degradation pathways (**S3–S7 Figs**). Combined with the representative functional pathways mentioned, we inferred that these genera may be responsible for the removal of phenols, CN^−^, and degradable aliphatic hydrocarbon (**S8 Fig**). Because of the high removal rates of phenols, CN^−^, and COD in the bioreactor, the identified major genera may contribute in the degradation of these pollutants and in reducing the toxicity of coking wastewater.

## Conclusions

We characterized the diversity and function of the microbial communities involved in the first aerobic bioreactor of an O/H/O coking wastewater treatment system. Proteobacteria, Bacteroidetes, and Chlorobi constituted the main decarbonization and detoxification phyla in the prefix aerobic bioreactor, with β-Proteobacteria and α-Proteobacteria existing, unexpectedly, as the most abundant classes. Compared to the first anaerobic bioreactor of the traditional A/O/O process, γ-Proteobacteria was the predominant class. Our results showed that the community structure was significantly different between the first aerobic bioreactor and the anaerobic bioreactor. *Thiobacillus*, *Rhodoplanes*, *Lysobacter*, and *Leucobacter* were the major genera and might be responsible for decarbonization. Functional genes involved in the degradation of benzoate, phenyl cyanide, naphthalene, and vinyl cyanide were enriched. The dominant bacterial communities may facilitate pollutant degradation in the first aerobic bioreactor. Our results provide a speculated evidence for the refinement of the O/H/O systems and for the development of efficient strategies for coking wastewater treatment. In the future study, we would focus on controlling the threshold of complete ammonification/partial nitrification/complete nitrification, performed by microbes in the first aerobic bioreactor of coking wastewater treatment.

## Supporting information

S1 Fig Rarefaction curves of activated sludge samples. Operational taxonomic units (OTUs) were defined at a dissimilarity level of 3% in the 16S rRNA gene sequences. N: First aerobic bioreactor of the north sample; S: First aerobic bioreactor of the south sample.
(TIFF)

S2 Fig Relative abundance of the orders/families in the first aerobic bioreactors. Orders/families with relative abundance > 1% at least one of samples were defined as major orders/families, and orders/families that accounted for a proportion of < 1% were defined as minor order/family. The average values of the three samples were calculated to represent the value of the corresponding sample. Refer to Table 1 for N and S. (a) Order; (b) Family.
(TIF)

S3 Fig Benzoate degradation pathways in the first aerobic bioreactor were predicted by KEGG pathway database. Different color cell represent different ranges of RA (relative abundance): Magenta RA ≥ 1%; orange 0.1% ≤ RA < 1%; yellow 0.01% ≤ RA <0.1%; green 0.001% ≤ RA < 0.01%; blue RA < 0.001%; and white cell indicate that some biodegradable pathways have not been found.
(PDF)

S4 Fig Aminobenzoate degradation pathways in the first aerobic bioreactor were predicted by KEGG pathway database. Refer to S3 Fig for different color cell. The dotted arrow indicate that some biodegradable pathways have not been found.
(PDF)

S5 Fig Caprolactam degradation pathways in the first aerobic bioreactor were predicted by KEGG pathway database. Refer to S3 Fig for different color cell.
(PDF)

S6 Fig Naphthalene degradation pathways in the first aerobic bioreactor were predicted by KEGG pathway database. Refer to S3 Fig for different color cell and refer to S4 Fig for the dotted arrow.
(PDF)

S7 Fig Styrene degradation pathways in the first aerobic bioreactor were predicted by KEGG pathway database. Refer to S3 Fig for different color cell.
(PDF)

S8 Fig RDA based on major genera and environmental parameters. Red arrows: Major genera; blue arrows: Environmental parameters.
(PDF)

S1 Table Relative abundance (%) of the 16S rRNA MiSeq gene sequences retrieved from the first aerobic bioreactor assigned to different phyla. (DOCX)

S2 Table The relative abundance (%) of dominant orders and families in the first aerobic bioreactor of north and south subsystem. (DOCX)

S3 Table Sequences assignment results at genus level. (XLSX)

S4 Table The relative abundance of top 21 OTUs in the first aerobic bioreactor of north and south subsystem. (DOCX)

S5 Table The enzyme and xenobiotic degradation and metabolism pathways involved in major genera. (DOCX)

## References

[1] Pan J, Ma J, Wu H, Chen B, He M, Liao C, et al Application of metabolic division of labor in simultaneous removal of nitrogen and thiocyanate from wastewater. Water Res. 2019;150:216–24. Epub 2018/12/12. 10.1016/j.watres.2018.11.070 . 30528918

[2] Zhang WH, Wei CH, Chai XS, He JY, Cai Y, Ren M, et al The behaviors and fate of polycyclic aromatic hydrocarbons (PAHs) in a coking wastewater treatment plant. Chemosphere. 2012;88(2):174–82. Epub 2012/04/03. 10.1016/j.chemosphere.2012.02.076 . 22464861

[3] Zhao WT, Sui Q, Huang X. Removal and fate of polycyclic aromatic hydrocarbons in a hybrid anaerobic-anoxic-oxic process for highly toxic coke wastewater treatment. Sci Total Environ. 2018;635:716–24. Epub 2018/04/24. 10.1016/j.scitotenv.2018.04.162 . 29680762

[4] Ma JD, Wu HZ, Wang YX, Qiu GL, Fu BB, Wu CF, et al Material inter-recycling for advanced nitrogen and residual COD removal from bio-treated coking wastewater through autotrophic denitrification. Bioresour Technol. 2019;289:121616 Epub 2019/06/22. 10.1016/j.biortech.2019.121616 . 31226671

[5] Ofman P, Struk-Sokolowska J, Skoczko I, Wiater J. Alternated biodegradation of naphthalene (NAP), acenaphthylene (ACY) and acenaphthene (ACE) in an aerobic granular sludge reactor (GSBR). J Hazard Mater. 2020;383:121184 Epub 2019/09/16. 10.1016/j.jhazmat.2019.121184 . 31522063

[6] Zhu S, Wu HZ, Wu CF, Qiu GL, Feng CH, Wei CH. Structure and function of microbial community involved in a novel full-scale prefix oxic coking wastewater treatment O/H/O system. Water Res. 2019;164:114963 Epub 2019/08/20. 10.1016/j.watres.2019.114963 31421512

[7] Joshi DR, Zhang Y, Gao Y, Liu Y, Yang M. Biotransformation of nitrogen- and sulfur-containing pollutants during coking wastewater treatment: Correspondence of performance to microbial community functional structure. Water Res. 2017;121:338–48. Epub 2017/06/02. 10.1016/j.watres.2017.05.045 . 28570873

[8] Manavi N, Kazemi AS, Bonakdarpour B. The development of aerobic granules from conventional activated sludge under anaerobic-aerobic cycles and their adaptation for treatment of dyeing wastewater. Chemical Engineering Journal. 2017;312:375–84. 10.1016/j.cej.2016.11.155

[9] Wu HZ, Wang M, Zhu S, Xie JT, Preis S, Li FS, et al Structure and function of microbial community associated with phenol co-substrate in degradation of benzo[a]pyrene in coking wastewater. Chemosphere. 2019;228:128–38. Epub 2019/04/29. 10.1016/j.chemosphere.2019.04.117 . 31029958

[10] Kim YM, Cho HU, Lee DS, Park C, Park D, Park JM. Response of nitrifying bacterial communities to the increased thiocyanate concentration in pre-denitrification process. Bioresour Technol. 2011;102(2):913–22. Epub 2010/10/12. 10.1016/j.biortech.2010.09.032 . 20933392

[11] Wei CH, Xie B, Xiao HL, Wang DS. Volumetric mass transfer coefficient of oxygen in an internal loop airlift reactor with a convergence-divergence draft tube. Chem Eng Technol. 2000;23(7):597–603. 10.1002/1521-4125(200007)23:7&lt;597::Aid-Ceat597&gt;3.0.Co;2-Y WOS:000088286500008.

[12] Zhang T, Wei C, Feng C, Zhu J. A novel airlift reactor enhanced by funnel internals and hydrodynamics prediction by the CFD method. Bioresour Technol. 2012;104:600–7. Epub 2011/11/29. 10.1016/j.biortech.2011.11.008 . 22119313

[13] Zhu S, Wu HZ, Wei CH, Zhou L, Xie JT. Contrasting microbial community composition and function perspective in sections of a full-scale coking wastewater treatment system. Appl Microbiol Biotechnol. 2016;100(2):949–60. Epub 2015/10/03. 10.1007/s00253-015-7009-z . 26428241

[14] Bates ST, Berg-Lyons D, Caporaso JG, Walters WA, Knight R, Fierer N. Examining the global distribution of dominant archaeal populations in soil. ISME J. 2011;5(5):908–17. Epub 2010/11/19. 10.1038/ismej.2010.171 21085198 PMC3105767

[15] Zou B, Li JF, Zhou Q, Quan ZX. MIPE: A metagenome-based community structure explorer and SSU primer evaluation tool. PLoS One. 2017;12(3):e0174609 Epub 2017/03/30. 10.1371/journal.pone.0174609 28350876 PMC5370157

[16] Schloss PD, Westcott SL, Ryabin T, Hall JR, Hartmann M, Hollister EB, et al Introducing mothur: open-source, platform-independent, community-supported software for describing and comparing microbial communities. Appl Environ Microbiol. 2009;75(23):7537–41. Epub 2009/10/06. 10.1128/AEM.01541-09 19801464 PMC2786419

[17] Caporaso JG, Kuczynski J, Stombaugh J, Bittinger K, Bushman FD, Costello EK, et al QIIME allows analysis of high-throughput community sequencing data. Nat Methods. 2010;7(5):335–6. Epub 2010/04/13. 10.1038/nmeth.f.303 20383131 PMC3156573

[18] Langille MG, Zaneveld J, Caporaso JG, McDonald D, Knights D, Reyes JA, et al Predictive functional profiling of microbial communities using 16S rRNA marker gene sequences. Nat Biotechnol. 2013;31(9):814–21. Epub 2013/08/27. 10.1038/nbt.2676 23975157 PMC3819121

[19] McDonald D, Price MN, Goodrich J, Nawrocki EP, DeSantis TZ, Probst A, et al An improved Greengenes taxonomy with explicit ranks for ecological and evolutionary analyses of bacteria and archaea. ISME J. 2012;6(3):610–8. Epub 2011/12/03. 10.1038/ismej.2011.139 22134646 PMC3280142

[20] Sahariah BP, Anandkumar J, Chakraborty S. Treatment of coke oven wastewater in an anaerobic–anoxic–aerobic moving bed bioreactor system. Desalination and Water Treatment. 2015;57(31):14396–402. 10.1080/19443994.2015.1065448

[21] Cipollone R, Frangipani E, Tiburzi F, Imperi F, Ascenzi P, Visca P. Involvement of Pseudomonas aeruginosa rhodanese in protection from cyanide toxicity. Appl Environ Microbiol. 2007;73(2):390–8. Epub 2006/11/14. 10.1128/AEM.02143-06 17098912 PMC1796984

[22] Staib C, Lant P. Thiocyanate degradation during activated sludge treatment of coke-ovens wastewater. Biochemical Engineering Journal. 2007;34(2):122–30. 10.1016/j.bej.2006.11.029 WOS:000245828500004.

[23] Luque-Almagro VM, Cabello P, Saez LP, Olaya-Abril A, Moreno-Vivian C, Roldan MD. Exploring anaerobic environments for cyanide and cyano-derivatives microbial degradation. Appl Microbiol Biotechnol. 2018;102(3):1067–74. Epub 2017/12/07. 10.1007/s00253-017-8678-6 29209795 PMC5778177

[24] Kim YM, Park H, Cho KH, Park JM. Long term assessment of factors affecting nitrifying bacteria communities and N-removal in a full-scale biological process treating high strength hazardous wastewater. Bioresour Technol. 2013;134:180–9. Epub 2013/03/19. 10.1016/j.biortech.2013.02.036 . 23500576

[25] Na C, Zhang Y, Quan X, Chen S, Liu W, Zhang Y. Evaluation of the detoxification efficiencies of coking wastewater treated by combined anaerobic-anoxic-oxic (A(2)O) and advanced oxidation process. J Hazard Mater. 2017;338:186–93. Epub 2017/05/30. 10.1016/j.jhazmat.2017.05.037 . 28554110

[26] Wang W, Ma W, Han H, Li H, Yuan M. Thermophilic anaerobic digestion of Lurgi coal gasification wastewater in a UASB reactor. Bioresour Technol. 2011;102(3):2441–7. Epub 2010/11/30. 10.1016/j.biortech.2010.10.140 . 21112778

[27] Joshi DR, Zhang Y, Tian Z, Gao YX, Yang M. Performance and microbial community composition in a long-term sequential anaerobic-aerobic bioreactor operation treating coking wastewater. Appl Microbiol Biotechnol. 2016;100(18):8191–202. Epub 2016/05/26. 10.1007/s00253-016-7591-8 . 27221291

[28] Zhou S, Wei C, Liao C, Wu H. Comprehensive study on dynamics of microbial community in Anaerobic-Oxic-Oxic process using PCR-DGGE, gas chromatography analysis, and dehydrogenase activity assays. World Journal of Microbiology and Biotechnology. 2009;26(2):273–9. 10.1007/s11274-009-0170-8

[29] Zhao WT, Huang X, Lee DJ. Enhanced treatment of coke plant wastewater using an anaerobic-anoxic-oxic membrane bioreactor system. Separation and Purification Technology. 2009;66(2):279–86. 10.1016/j.seppur.2008.12.028 WOS:000265515700010.

[30] Wang S, Hou WG, Dong HL, Jiang HC, Huang LQ, Wu G, et al Control of temperature on microbial community structure in hot springs of the Tibetan Plateau. PLoS One. 2013;8(5):e62901 Epub 2013/05/15. 10.1371/journal.pone.0062901 23667538 PMC3647046

[31] Zhang XH, Tang S, Wang M, Sun WM, Xie YW, Peng H, et al Acid mine drainage affects the diversity and metal resistance gene profile of sediment bacterial community along a river. Chemosphere. 2019;217:790–9. Epub 2018/11/20. 10.1016/j.chemosphere.2018.10.210 . 30453276

[32] Wu LW, Ning DL, Zhang B, Li Y, Zhang P, Shan XY, et al Global diversity and biogeography of bacterial communities in wastewater treatment plants. Nat Microbiol. 2019;4(7):1183–95. Epub 2019/05/16. 10.1038/s41564-019-0426-5 . 31086312

[33] Qiu GL, Zhang S, Srinivasa Raghavan DS, Das S, Ting YP. Towards high through-put biological treatment of municipal wastewater and enhanced phosphorus recovery using a hybrid microfiltration-forward osmosis membrane bioreactor with hydraulic retention time in sub-hour level. Bioresour Technol. 2016;219:298–310. Epub 2016/08/09. 10.1016/j.biortech.2016.07.126 . 27498011

[34] Kaiser K, Wemheuer B, Korolkow V, Wemheuer F, Nacke H, Schoning I, et al Driving forces of soil bacterial community structure, diversity, and function in temperate grasslands and forests. Sci Rep. 2016;6:33696 Epub 2016/09/22. 10.1038/srep33696 27650273 PMC5030646

[35] Wang Q, Yang M, Song X, Tang S, Yu L. Aerobic and Anaerobic Biodegradation of 1,2-Dibromoethane by a Microbial Consortium under Simulated Groundwater Conditions. Int J Environ Res Public Health. 2019;16(19). Epub 2019/10/11. 10.3390/ijerph16193775 31597267 PMC6802363

[36] Cydzik-Kwiatkowska A, Zielinska M. Bacterial communities in full-scale wastewater treatment systems. World J Microbiol Biotechnol. 2016;32(4):66 Epub 2016/03/05. 10.1007/s11274-016-2012-9 26931606 PMC4773473

[37] Zhou J, Li HS, Chen XL, Wan DJ, Mai WN, Sun CQ. Cometabolic degradation of low-strength coking wastewater and the bacterial community revealed by high-throughput sequencing. Bioresour Technol. 2017;245(Pt A):379–85. Epub 2017/09/13. 10.1016/j.biortech.2017.08.119 . 28898834

[38] Zhu XB, Tian JP, Liu C, Chen LJ. Composition and dynamics of microbial community in a zeolite biofilter-membrane bioreactor treating coking wastewater. Appl Microbiol Biotechnol. 2013;97(19):8767–75. Epub 2012/12/12. 10.1007/s00253-012-4558-2 . 23229568

[39] Dyksma S, Lenk S, Sawicka JE, Mussmann M. Uncultured Gammaproteobacteria and Desulfobacteraceae Account for Major Acetate Assimilation in a Coastal Marine Sediment. Front Microbiol. 2018;9:3124 Epub 2019/01/09. 10.3389/fmicb.2018.03124 30619197 PMC6305295

[40] Yang Q, Xiong PP, Ding PY, Chu LB, Wang JL. Treatment of petrochemical wastewater by microaerobic hydrolysis and anoxic/oxic processes and analysis of bacterial diversity. Bioresour Technol. 2015;196:169–75. Epub 2015/08/04. 10.1016/j.biortech.2015.07.087 . 26233329

[41] Zhao LM, Song TW, Han D, Bao MT, Lu JR. Hydrolyzed polyacrylamide biotransformation in an up-flow anaerobic sludge blanket reactor system: key enzymes, functional microorganisms, and biodegradation mechanisms. Bioprocess Biosyst Eng. 2019;42(6):941–51. Epub 2019/03/02. 10.1007/s00449-019-02094-w . 30820666

[42] Chen B, Yang Z, Pan JX, Ren Y, Wu HZ, Wei CH. Functional identification behind gravity-separated sludge in high concentration organic coking wastewater: Microbial aggregation, apoptosis-like decay and community. Water Res. 2019;150:120–8. Epub 2018/12/06. 10.1016/j.watres.2018.11.040 . 30508709

[43] Ma Q, Qu YY, Shen WL, Zhang ZJ, Wang JW, Liu ZY, et al Bacterial community compositions of coking wastewater treatment plants in steel industry revealed by Illumina high-throughput sequencing. Bioresour Technol. 2015;179:436–43. Epub 2015/01/09. 10.1016/j.biortech.2014.12.041 . 25569032

[44] Srinivas A, Sasikala C, Ramana CHV. Rhodoplanes oryzae sp. nov., a phototrophic alphaproteobacterium isolated from the rhizosphere soil of paddy. Int J Syst Evol Microbiol. 2014;64(Pt 7):2198–203. Epub 2014/04/04. 10.1099/ijs.0.063347-0 . 24695055

[45] Reis AC, Cvancarova M, Liu Y, Lenz M, Hettich T, Kolvenbach BA, et al Biodegradation of sulfamethoxazole by a bacterial consortium of Achromobacter denitrificans PR1 and Leucobacter sp. GP. Appl Microbiol Biotechnol. 2018;102(23):10299–314. Epub 2018/10/09. 10.1007/s00253-018-9411-9 . 30294753

[46] Zhao ZM, Xu CL, Zhang X, Song XS. Addition of iron materials for improving the removal efficiencies of multiple contaminants from wastewater with a low C/N ratio in constructed wetlands at low temperatures. Environ Sci Pollut Res Int. 2019;26(12):11988–97. Epub 2019/03/04. 10.1007/s11356-019-04648-7 . 30827018

[47] Zhang DD, Cui L, Wang H, Liang JY. Study of sulfate-reducing ammonium oxidation process and its microbial community composition. Water Sci Technol. 2019;79(1):137–44. Epub 2019/03/01. 10.2166/wst.2019.027 ; PubMed Central PMCID: PMCwst_2019_027. 30816870

[48] Law YY, Matysik A, Chen XM, Swa Thi S, Ngoc Nguyen TQ, Qiu GL, et al High Dissolved Oxygen Selection against Nitrospira Sublineage I in Full-Scale Activated Sludge. Environ Sci Technol. 2019;53(14):8157–66. Epub 2019/06/12. 10.1021/acs.est.9b00955 . 31184114

[49] Tian HL, Hui M, Pan PP, Huang JH, Chen L, Zhao JY. Performance and microbial ecology of biofilms adhering on aerated membrane with distinctive conditions for the treatment of domestic sewage. Environ Technol. 2019:1–9. Epub 2019/06/18. 10.1080/09593330.2019.1631890 . 31204896

[50] Zhu S, Wu H, Zhou L, Wei C. The Resilience of Microbial Community Involved in Coking Wastewater Treatment System. Journal of Next Generation Sequencing & Applications. 2017;04(01). 10.4172/2469-9853.1000142

[51] Manefield M, Griffiths RI, Leigh MB, Fisher R, Whiteley AS. Functional and compositional comparison of two activated sludge communities remediating coking effluent. Environ Microbiol. 2005;7(5):715–22. Epub 2005/04/12. 10.1111/j.1462-2920.2004.00746.x . 15819853

[52] Pumphrey GM, Madsen EL. Field-based stable isotope probing reveals the identities of benzoic acid-metabolizing microorganisms and their in situ growth in agricultural soil. Appl Environ Microbiol. 2008;74(13):4111–8. Epub 2008/05/13. 10.1128/AEM.00464-08 18469130 PMC2446519

[53] Singleton DR, Powell SN, Sangaiah R, Gold A, Ball LM, Aitken MD. Stable-isotope probing of bacteria capable of degrading salicylate, naphthalene, or phenanthrene in a bioreactor treating contaminated soil. Appl Environ Microbiol. 2005;71(3):1202–9. Epub 2005/03/05. 10.1128/AEM.71.3.1202-1209.2005 15746319 PMC1065189

[54] Sun WM, Xie SG, Luo CL, Cupples AM. Direct link between toluene degradation in contaminated-site microcosms and a Polaromonas strain. Appl Environ Microbiol. 2010;76(3):956–9. Epub 2009/12/17. 10.1128/AEM.01364-09 20008173 PMC2813006

[55] Shi SN, Zhang XW, Ma F, Sun TH, Li A, Zhou JT, et al Cometabolic degradation of dibenzofuran by Comamonas sp MQ. Process Biochemistry. 2013;48(10):1553–8. 10.1016/j.procbio.2013.07.003 WOS:000325196900014.

[56] Ni B, Zhang Y, Chen DW, Wang BJ, Liu SJ. Assimilation of aromatic compounds by Comamonas testosteroni: characterization and spreadability of protocatechuate 4,5-cleavage pathway in bacteria. Appl Microbiol Biotechnol. 2013;97(13):6031–41. Epub 2012/09/22. 10.1007/s00253-012-4402-8 . 22996279

[57] Xu WC, Zhang YX, Cao HB, Sheng YX, Li HB, Li YP, et al Metagenomic insights into the microbiota profiles and bioaugmentation mechanism of organics removal in coal gasification wastewater in an anaerobic/anoxic/oxic system by methanol. Bioresour Technol. 2018;264:106–15. Epub 2018/05/25. 10.1016/j.biortech.2018.05.064 . 29793117

[58] Holmes B, Steigerwalt A, Weaver R, Brenner D. Weeksella Bergey's Manual of Systematics of Archaea and Bacteria, John Wiley & Sons, Inc,. 2015 10.1002/9781118960608.gbm00349

[59] Chen R, Yao J, Ailijiang N, Liu R, Fang L, Chen Y. Abundance and diversity of nitrogen-removing microorganisms in the UASB-anammox reactor. PLoS One. 2019;14(4):e0215615 Epub 2019/04/23. 10.1371/journal.pone.0215615 31009503 PMC6476503

[60] Antwi P, Zhang DC, Luo WH, Xiao LW, Meng J, Kabutey FT, et al Performance, microbial community evolution and neural network modeling of single-stage nitrogen removal by partial-nitritation/anammox process. Bioresour Technol. 2019;284:359–72. Epub 2019/04/08. 10.1016/j.biortech.2019.03.008 . 30954904

[61] Li Y, Huang ZX, Ruan WQ, Ren HY, Zhao MX. ANAMMOX performance, granulation, and microbial response under COD disturbance. Journal of Chemical Technology & Biotechnology. 2015;90(1):139–48. 10.1002/jctb.4298

[62] Miao Y, Zhang L, Li B, Zhang Q, Wang S, Peng Y. Enhancing ammonium oxidizing bacteria activity was key to single-stage partial nitrification-anammox system treating low-strength sewage under intermittent aeration condition. Bioresour Technol. 2017;231:36–44. Epub 2017/02/14. 10.1016/j.biortech.2017.01.045 . 28192724

[63] Niu T, Zhou Z, Shen X, Qiao W, Jiang LM, Pan W, et al Effects of dissolved oxygen on performance and microbial community structure in a micro-aerobic hydrolysis sludge in situ reduction process. Water Res. 2016;90:369–77. Epub 2016/01/15. 10.1016/j.watres.2015.12.050 . 26766160

[64] Huang JL, Wang HH, Alam F, Cui YW. Granulation of halophilic sludge inoculated with estuarine sediments for saline wastewater treatment. Sci Total Environ. 2019;682:532–40. Epub 2019/05/28. 10.1016/j.scitotenv.2019.05.197 . 31129541

[65] Wang H, Li H-X, Fang F, Guo J-s, Chen Y-P, Yan P, et al Underlying mechanisms of ANAMMOX bacteria adaptation to salinity stress. Journal of Industrial Microbiology & Biotechnology. 2019;46(5):573–85. 10.1007/s10295-019-02137-x 30690673

[66] Balcom IN, Driscoll H, Vincent J, Leduc M. Metagenomic analysis of an ecological wastewater treatment plant's microbial communities and their potential to metabolize pharmaceuticals. F1000Res. 2016;5:1881 Epub 2016/09/10. 10.12688/f1000research.9157.1 27610223 PMC4995686

[67] Zafra G, Taylor TD, Absalon AE, Cortes-Espinosa DV. Comparative metagenomic analysis of PAH degradation in soil by a mixed microbial consortium. J Hazard Mater. 2016;318:702–10. Epub 2016/08/04. 10.1016/j.jhazmat.2016.07.060 . 27484946

[68] Joshi DR, Zhang Y, Zhang H, Gao YX, Yang M. Characteristics of microbial community functional structure of a biological coking wastewater treatment system. J Environ Sci (China). 2018;63:105–15. Epub 2018/02/07. 10.1016/j.jes.2017.07.011 . 29406094

