# Supplementary figures and images for: Diversity and functional prediction of microbial communities involved in the first aerobic bioreactor of coking wastewater treatment system
Source: PLoS One. 2020 Dec 10;15(12):e0243748. doi: 10.1371/journal.pone.0243748 (PMC7728250; doi:10.1371/journal.pone.0243748)

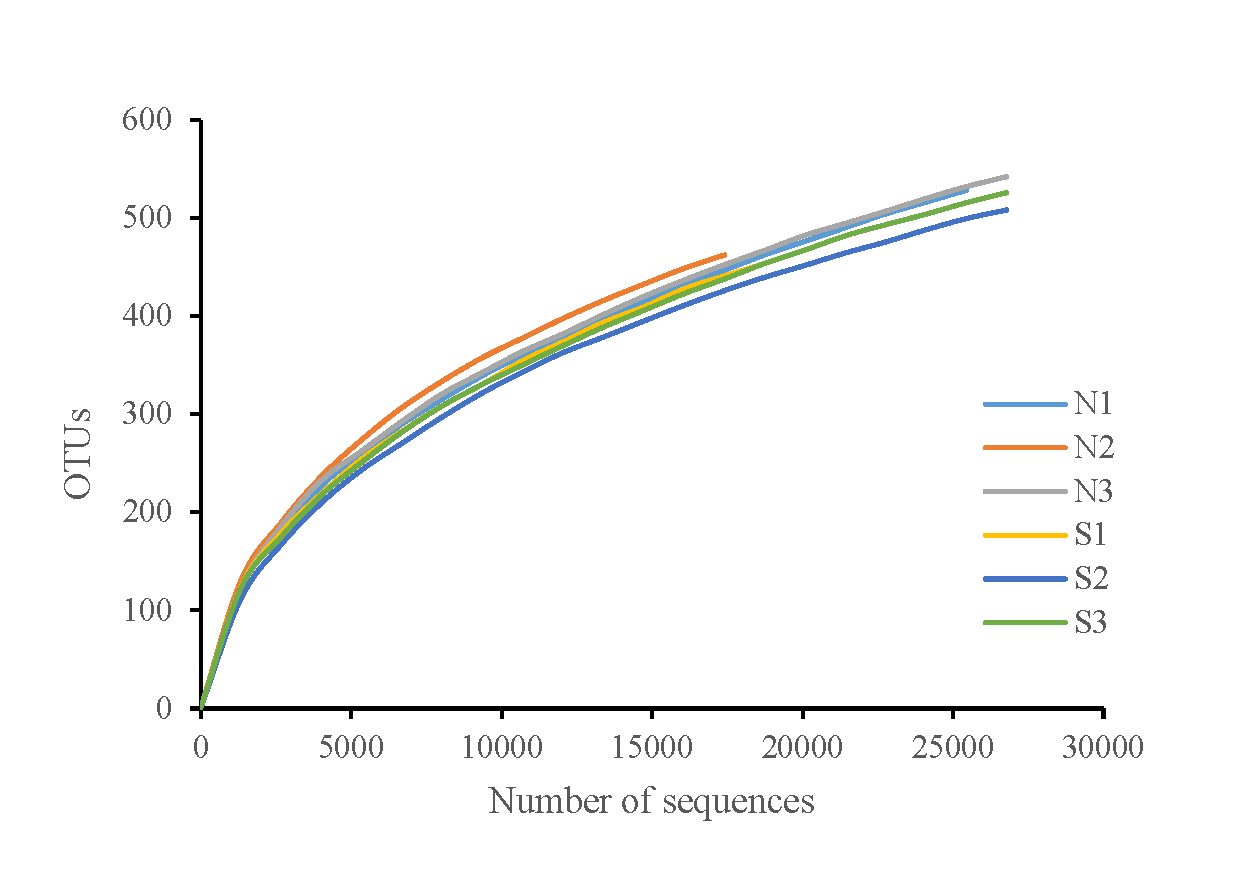

Supplement: S1 Fig — Operational taxonomic units (OTUs) were defined at a dissimilarity level of 3% in the 16S rRNA gene sequences. N: First aerobic bioreactor of the north sample; S: First aerobic bioreactor of the south sample. (TIFF) [file pone.0243748.s001.tiff]

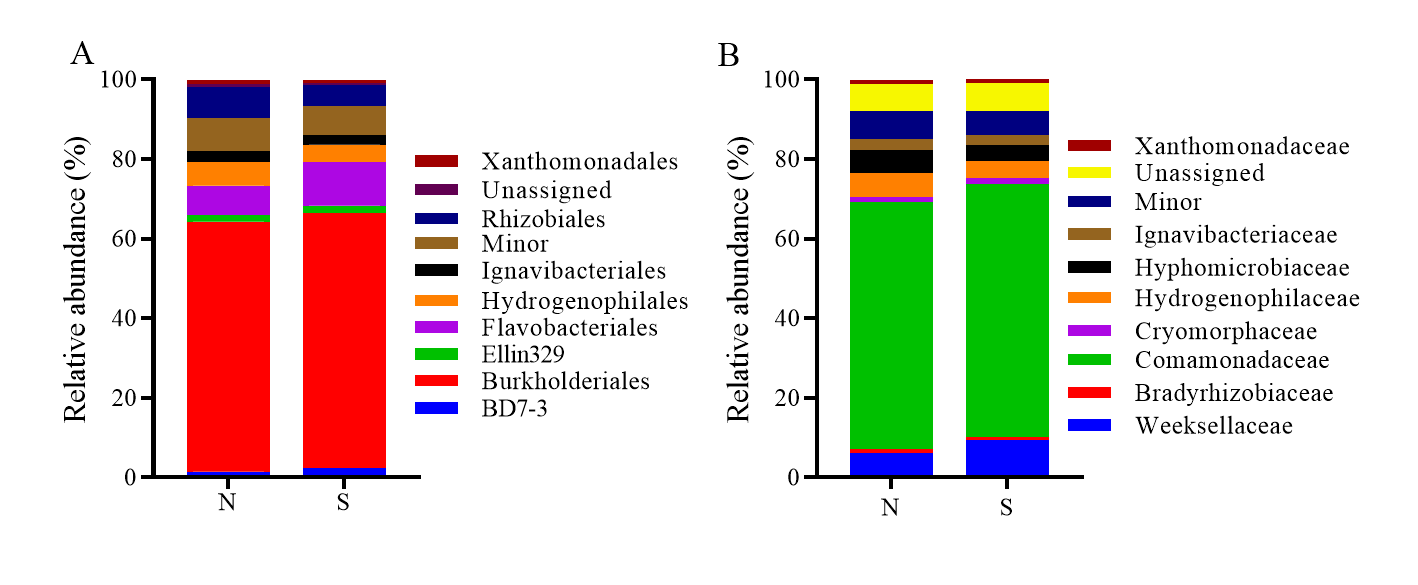

Supplement: S2 Fig — Orders/families with relative abundance > 1% at least one of samples were defined as major orders/families, and orders/families that accounted for a proportion of < 1% were defined as minor order/family. The average values of the three samples were calculated to represent the value of the corresponding sample. Refer to Table 1 for N and S. (a) Order; (b) Family. (TIF) [file pone.0243748.s002.tif]

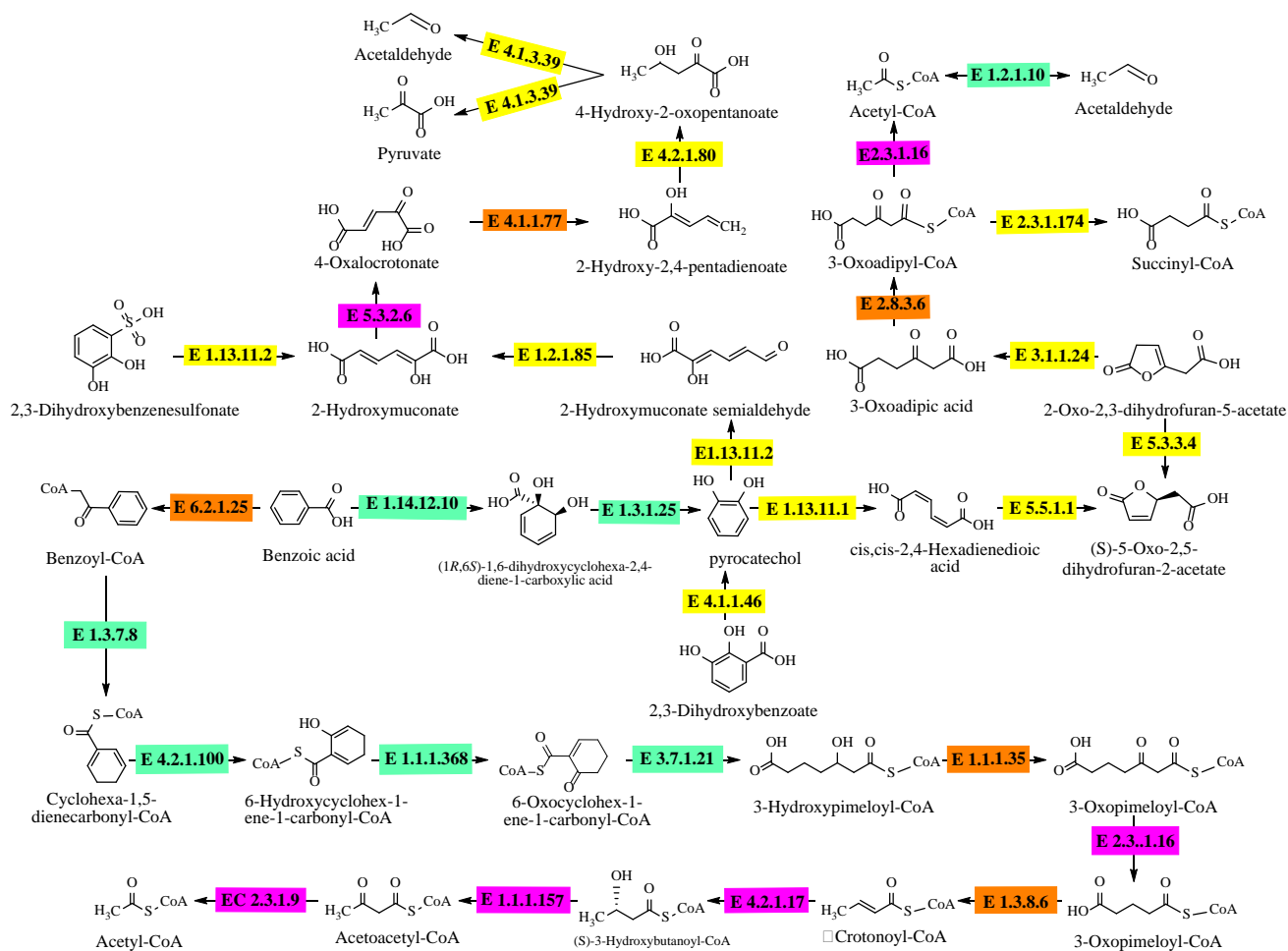

Supplement: S3 Fig — Different color cell represent different ranges of RA (relative abundance): Magenta RA ≥ 1%; orange 0.1% ≤ RA < 1%; yellow 0.01% ≤ RA <0.1%; green 0.001% ≤ RA < 0.01%; blue RA < 0.001%; and white cell indicate that some biodegradable pathways have not been found. (PDF) [file pone.0243748.s003.pdf]

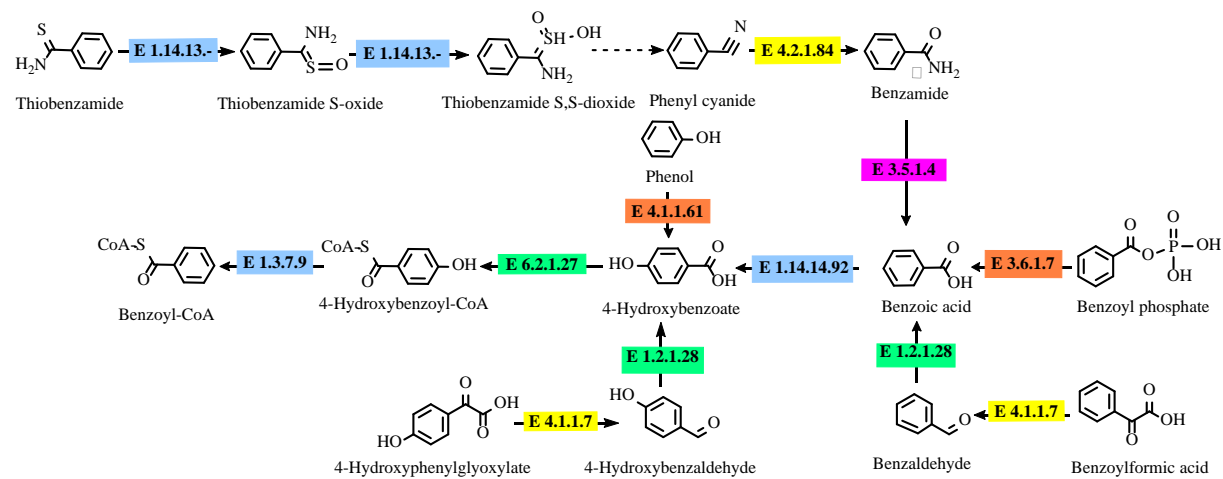

Supplement: S4 Fig — Refer to S3 Fig for different color cell. The dotted arrow indicate that some biodegradable pathways have not been found. (PDF) [file pone.0243748.s004.pdf]

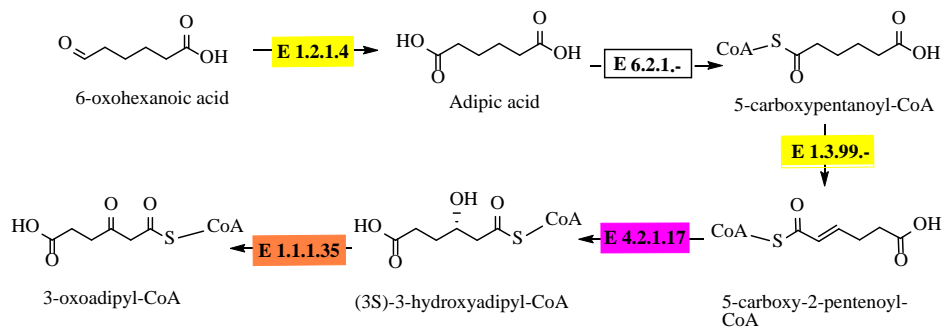

Supplement: S5 Fig — Refer to S3 Fig for different color cell. (PDF) [file pone.0243748.s005.pdf]

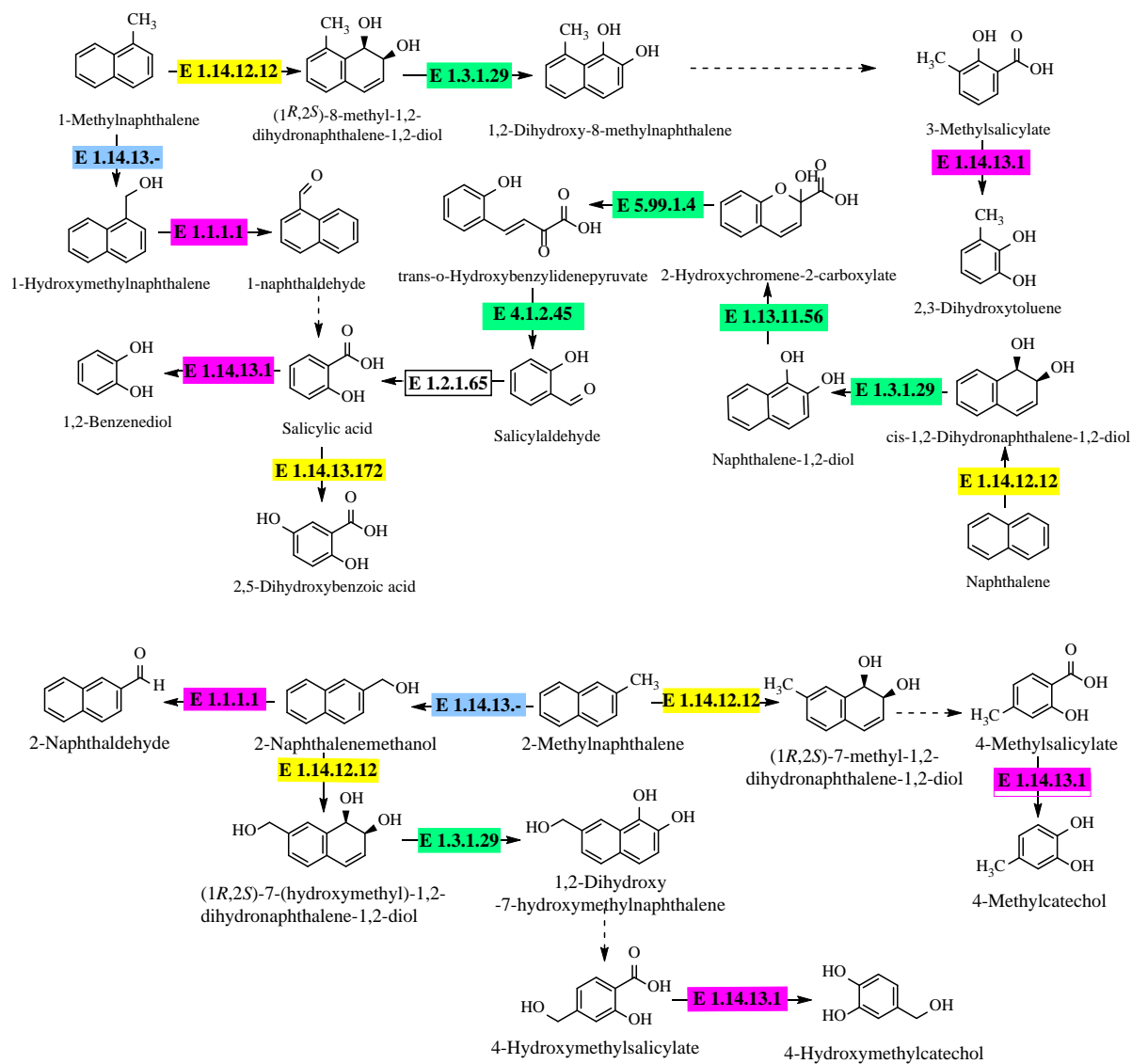

Supplement: S6 Fig — Refer to S3 Fig for different color cell and refer to S4 Fig for the dotted arrow. (PDF) [file pone.0243748.s006.pdf]

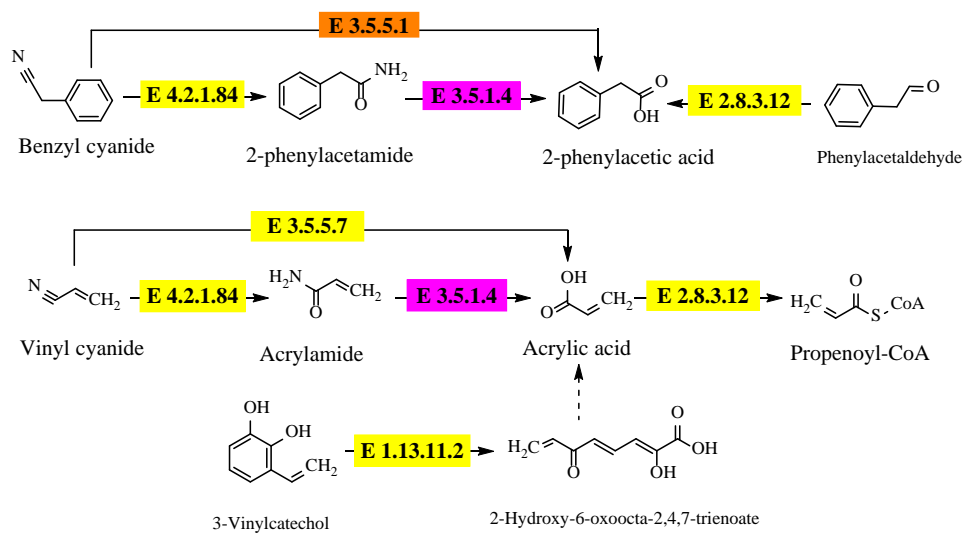

Supplement: S7 Fig — Refer to S3 Fig for different color cell. (PDF) [file pone.0243748.s007.pdf]

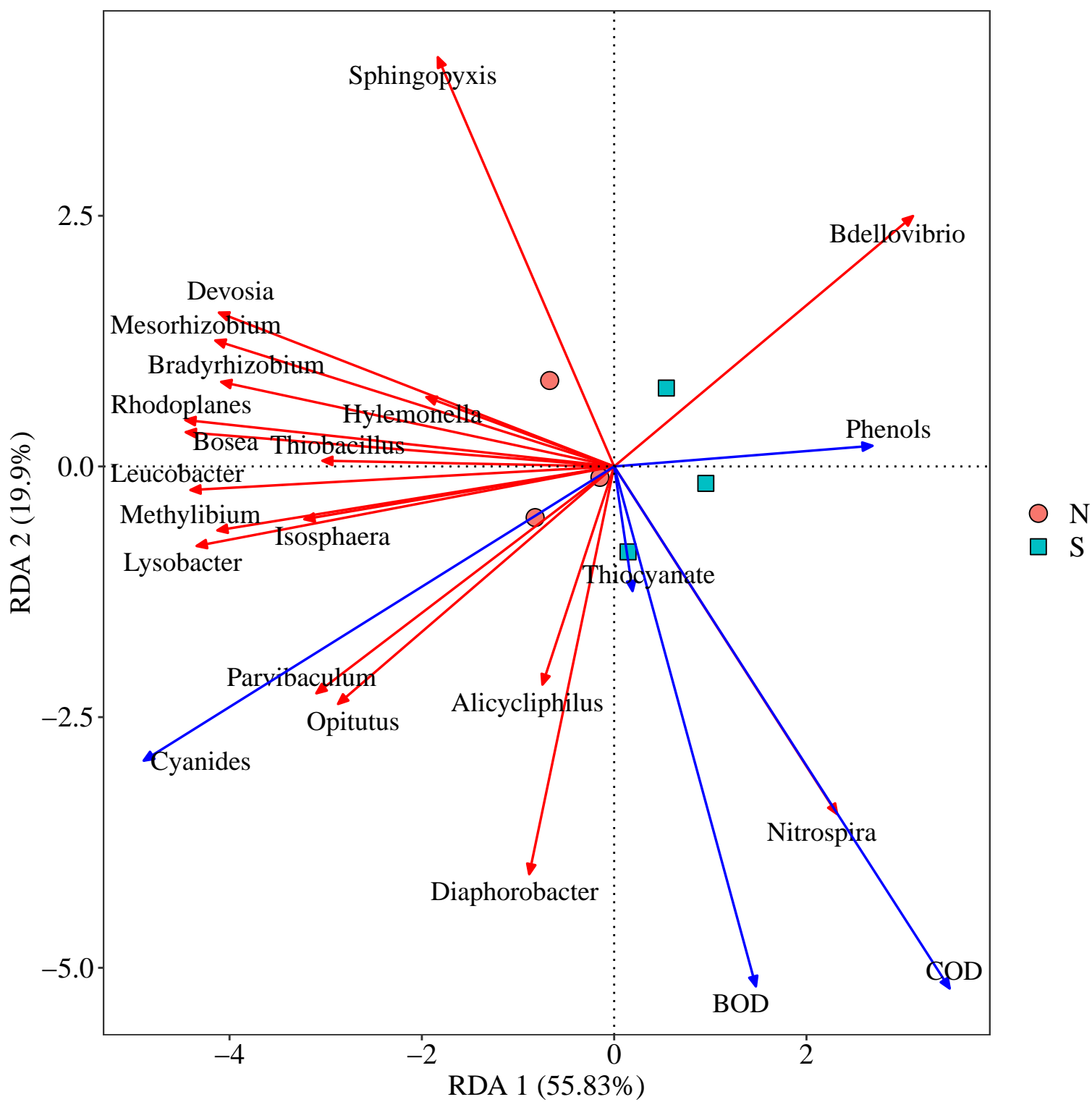

Supplement: S8 Fig — Red arrows: Major genera; blue arrows: Environmental parameters. (PDF) [file pone.0243748.s008.pdf]
