# Supplementary material for: Diversity and functional prediction of microbial communities involved in the first aerobic bioreactor of coking wastewater treatment system
Source: PLoS One. 2020 Dec 10;15(12):e0243748. doi: 10.1371/journal.pone.0243748 (PMC7728250; doi:10.1371/journal.pone.0243748)
Supplement: S1 Table — (DOCX) [file pone.0243748.s009.docx]

**S1 Table. Relative abundance (%) of the 16S rRNA MiSeq gene sequences retrieved from the first aerobic bioreactor assigned to different phyla.**

| **Sample ID** | Activated sludge of north | | |  | Activated sludge of south | | |
| --- | --- | --- | --- | --- | --- | --- | --- |
| **Phylum** | **N1** | **N2** | **N3** |  | **S1** | **S2** | **S3** |
| Bacteria |  |  |  |  |  |  |  |
| Acidobacteria | 0.72 | 0.87 | 0.80 |  | 0.76 | 0.76 | 0.72 |
| Actinobacteria | 0.99 | **1.23** | 0.61 |  | 0.41 | 0.24 | 0.55 |
| Armatimonadetes | 0.00 | 0.00 | 0.00 |  | 0.01 | 0.00 | 0.00 |
| Bacteroidetes | **9.26** | **4.58** | **11.13** |  | **14.24** | **17.20** | **5.36** |
| Chlamydiae | 0.02 | 0.02 | 0.02 |  | 0.01 | 0.03 | 0.01 |
| Chlorobi | **2.20** | **2.05** | **4.07** |  | **3.42** | **1.84** | **2.18** |
| Chloroflexi | 0.12 | 0.22 | 0.07 |  | 0.05 | 0.03 | 0.14 |
| Cyanobacteria | 0.00 | 0.00 | 0.02 |  | 0.01 | 0.01 | 0.00 |
| Firmicutes | 0.21 | 0.24 | 0.18 |  | 0.16 | 0.22 | 0.18 |
| Gemmatimonadetes | 0.02 | 0.03 | 0.03 |  | 0.01 | 0.05 | 0.03 |
| Nitrospirae | 0.30 | 0.33 | 0.36 |  | 0.31 | 0.38 | 0.37 |
| NKB19 | 0.00 | 0.00 | 0.00 |  | 0.00 | 0.00 | 0.00 |
| OP8 | 0.00 | 0.01 | 0.01 |  | 0.00 | 0.00 | 0.00 |
| Planctomycetes | 0.73 | **1.25** | 0.99 |  | 0.70 | 0.39 | 0.65 |
| Proteobacteria | **85.05** | **88.71** | **81.09** |  | **79.64** | **78.62** | **89.52** |
| Alphaproteobacteria | **11.95** | **11.29** | **12.36** |  | **11.79** | **9.72** | **9.72** |
| Betaproteobacteria | **69.09** | **73.10** | **65.05** |  | **64.49** | **65.78** | **75.98** |
| Deltaproteobacteria | 0.17 | 0.15 | 0.22 |  | 0.32 | 0.22 | 0.17 |
| Epsilonproteobacteria | 0.04 | 0.04 | 0.04 |  | 0.05 | 0.04 | 0.04 |
| Gammaproteobacteria | **3.81** | **4.12** | **3.41** |  | **2.99** | **2.85** | **3.60** |
| Unclassified Proteobacteria | 0.00 | 0.01 | 0.00 |  | 0.00 | 0.00 | 0.00 |
| SBR1093 | 0.16 | 0.16 | 0.33 |  | 0.09 | 0.08 | 0.14 |
| Spirochaetes | 0.00 | 0.01 | 0.00 |  | 0.00 | 0.00 | 0.00 |
| Synergistetes | 0.02 | 0.01 | 0.00 |  | 0.01 | 0.01 | 0.00 |
| Tenericutes | 0.00 | 0.00 | 0.00 |  | 0.01 | 0.00 | 0.00 |
| Thermi | 0.03 | 0.03 | 0.01 |  | 0.01 | 0.00 | 0.01 |
| TM6 | 0.05 | 0.01 | 0.04 |  | 0.01 | 0.02 | 0.02 |
| Verrucomicrobia | 0.06 | 0.21 | 0.15 |  | 0.08 | 0.06 | 0.08 |
| WPS-2 | 0.05 | 0.05 | 0.07 |  | 0.04 | 0.04 | 0.03 |
| Unclassified bacteria | 0.01 | 0.01 | 0.00 |  | 0.02 | 0.01 | 0.00 |
| Unclassified to domain | 0.00 | 0.00 | 0.00 |  | 0.00 | 0.00 | 0.00 |

Sequences were assigned using RDP Classifier at a confidence threshold of 80%. Refer to Table 2 for sample abbreviations.
