# Supplementary material for: Diversity and functional prediction of microbial communities involved in the first aerobic bioreactor of coking wastewater treatment system
Source: PLoS One. 2020 Dec 10;15(12):e0243748. doi: 10.1371/journal.pone.0243748 (PMC7728250; doi:10.1371/journal.pone.0243748)
Supplement: S2 Table — (DOCX) [file pone.0243748.s010.docx]

**S2 Table. The relative abundance (%) of dominant orders and families in the first aerobic bioreactor of north and south subsystem.**

| **Sample ID** | | | | Activated sludge of north | | |  | Activated sludge of south | | |
| --- | --- | --- | --- | --- | --- | --- | --- | --- | --- | --- |
| **Phylum** | **Class** | **Order** | **Family** | **N1** | **N2** | **N3** |  | **S1** | **S2** | **S3** |
| Bacteroidetes | Flavobacteriia | Flavobacteriales |  | 8.41 | 4.1 | 9.89 |  | 12.76 | 15.58 | 4.69 |
|  |  | Flavobacteriales | Weeksellaceae | 7.14 | 2.89 | 8.29 |  | 10.99 | 13.96 | 3.63 |
|  |  | Flavobacteriales | Cryomorphaceae | 1.27 | 1.21 | 1.6 |  | 1.78 | 1.62 | 1.05 |
| Chlorobi | Ignavibacteria | Ignavibacteriales |  | 2.2 | 2.05 | 4.07 |  | 3.42 | 1.84 | 2.18 |
|  |  | Ignavibacteriales | Ignavibacteriaceae | 2.2 | 2.05 | 4.07 |  | 3.42 | 1.84 | 2.18 |
| Proteobacteria | α-Pproteobacteria | BD7-3 |  | 1.38 | 0.95 | 2.21 |  | 2.97 | 2.97 | 1.08 |
|  |  | Ellin329 |  | 1.21 | 1.35 | 2.11 |  | 2.5 | 1.43 | 1.65 |
|  |  | Rhizobiales |  | 8.19 | 8 | 6.73 |  | 5.16 | 4.58 | 5.91 |
|  |  | Rhizobiales | Bradyrhizobiaceae | 1.17 | 0.95 | 0.91 |  | 0.65 | 0.63 | 0.84 |
|  |  | Rhizobiales | Hyphomicrobiaceae | 6.03 | 6.13 | 5.29 |  | 4.05 | 3.58 | 4.47 |
|  | β-Pproteobacteria | Burkholderiales |  | 63.12 | 67.33 | 58 |  | 59.9 | 61.3 | 71.06 |
|  |  | Burkholderiales | Comamonadaceae | 62.23 | 66.57 | 57.24 |  | 59.31 | 60.67 | 70.19 |
|  |  | Hydrogenophilales |  | 5.6 | 5.42 | 6.72 |  | 4.26 | 4.16 | 4.57 |
|  |  | Hydrogenophilales | Hydrogenophilaceae | 5.6 | 5.42 | 6.72 |  | 4.26 | 4.16 | 4.57 |
|  | γ-Pproteobacteria | Xanthomonadales |  | 1.2 | 1.39 | 1.05 |  | 0.88 | 0.85 | 1.03 |
|  |  | Xanthomonadales | Xanthomonadaceae | 1.18 | 1.39 | 1.04 |  | 0.88 | 0.83 | 1.02 |

Sequences were assigned using RDP Classifier at a confidence threshold of 80%. Refer to Table 2 for sample abbreviations. The relative abundance >1% in at least one of samples were defined as major order/family. The average values of the three samples were calculated to represent the value of the corresponding sample.
