# Supplementary material for: Diversity and functional prediction of microbial communities involved in the first aerobic bioreactor of coking wastewater treatment system
Source: PLoS One. 2020 Dec 10;15(12):e0243748. doi: 10.1371/journal.pone.0243748 (PMC7728250; doi:10.1371/journal.pone.0243748)
Supplement: S4 Table — (DOCX) [file pone.0243748.s012.docx]

**S4 Table. The relative abundance of top 21 OTUs in the first aerobic bioreactor of north and south subsystem.**

| **Phylum** | **Class** | **Order/family/genus/spicises_OTU.ID** | Activated sludge of north | | |  | Activated sludge of south | | |
| --- | --- | --- | --- | --- | --- | --- | --- | --- | --- |
|  |  |  | **N1** | **N2** | **N1** |  | **N2** | **N1** | **S3** |
| Acidobacteria | Chloracidobacteria | f_Ellin6075_4420696 | 0.54 | 0.55 | 0.47 |  | 0.49 | 0.56 | 0.41 |
| Actinobacteria | Actinobacteria | *g_Leucobacter*_1127702 | 0.72 | 0.88 | 0.43 |  | 0.24 | 0.15 | 0.38 |
| Bacteroidetes | Flavobacteriia | f_Weeksellaceae_4300908 | **5.82** | **2.21** | **6.63** |  | **8.74** | **11.33** | **2.95** |
|  |  | f_Weeksellaceae_4424932 | **1.07** | 0.51 | **1.3** |  | **1.71** | **2.12** | 0.47 |
|  |  | f_Cryomorphaceae_New.ReferenceOTU79 | **1.21** | **1.17** | **1.5** |  | **1.68** | **1.51** | 0.99 |
| Bacteroidetes | Saprospirae | f_Chitinophagaceae_843075 | 0.49 | 0.19 | 0.76 |  | 0.87 | **1.13** | 0.33 |
| Chlorobi | Ignavibacteria | f_Ignavibacteriaceae_589318 | **2.16** | **1.98** | **4.04** |  | **3.38** | **1.81** | **2.16** |
| Proteobacteria | α-Proteobacteria | o_BD7-3_890056 | **1.33** | 0.92 | **2.15** |  | **2.82** | **2.84** | **1.06** |
|  |  | o_Ellin329_New.ReferenceOTU34 | **1.15** | **1.31** | **2.02** |  | **2.43** | **1.37** | **1.57** |
|  |  | f_Bradyrhizobiaceae_826270 | 0.68 | 0.51 | 0.55 |  | 0.37 | 0.4 | 0.52 |
|  |  | *s_elegans*_275053 | **3.44** | **3.73** | **3.19** |  | **2.63** | **2.15** | **2.47** |
|  |  | *s_elegans*_544615 | 0.67 | 0.59 | 0.41 |  | 0.34 | 0.3 | 0.46 |
| Proteobacteria | β-Proteobacteria | f_Comamonadaceae_654788 | 0.62 | 0.61 | 0.49 |  | 0.55 | 0.56 | 0.61 |
|  |  | f_Comamonadaceae_838837 | 0.64 | 0.71 | 0.79 |  | 0.55 | 0.75 | 0.68 |
|  |  | f_Comamonadaceae_846710 | **57.33** | **61.23** | **52.48** |  | **54.57** | **55.78** | **64.8** |
|  |  | f_Comamonadaceae_940737 | 0.78 | 0.89 | 0.75 |  | 0.7 | 0.78 | 0.91 |
|  |  | *g_Thiobacillus*_850808 | **5.52** | **5.31** | **6.6** |  | **4.19** | **4.08** | **4.47** |
| Proteobacteria | γ-Proteobacteria | o_Chromatiales_New.ReferenceOTU39 | 0.62 | 0.56 | 0.62 |  | 0.58 | 0.37 | 0.58 |
|  |  | o_PYR10d3_2873181 | 0.9 | 0.95 | 0.76 |  | 0.47 | 0.46 | 0.94 |
|  |  | f_Pseudomonadaceae_312988 | 0.77 | 0.88 | 0.69 |  | 0.68 | 0.78 | 0.7 |
|  |  | *g_Lysobacter_1072787* | **1.05** | **1.16** | 0.89 |  | 0.74 | 0.74 | 0.9 |

Sequences were assigned using RDP Classifier at a confidence threshold of 80%. Refer to Table 2 for sample abbreviations.
