# Supplementary material for: Diversity and functional prediction of microbial communities involved in the first aerobic bioreactor of coking wastewater treatment system
Source: PLoS One. 2020 Dec 10;15(12):e0243748. doi: 10.1371/journal.pone.0243748 (PMC7728250; doi:10.1371/journal.pone.0243748)
Supplement: S5 Table — (DOCX) [file pone.0243748.s013.docx]

|  | *Leucobacter* | *Rhodoplanes* | *Lysobacter* |
| --- | --- | --- | --- |
| enoyl-CoA hydratase [E 4.2.1.17] |  | Chlorocyclohexane and chlorobenzene degradation, Fluorobenzoate degradation, Toluene degradation | |
| aldehyde dehydrogenase (NAD+) [EC 1.2.1.3] | Chloroalkane and chloroalkene degradation | | |
| acetyl-CoA C-acetyltransferase [E 2.3.1.9] | Benzoate degradation | | |
| amidase [E 3.5.1.4] | Aminobenzoate degradation, Styrene degradation | | |
| alcohol dehydrogenase [E 1.1.1.284 1.1.1.1] | Naphthalene degradation, Chloroalkane and chloroalkene degradation | | |
| carboxymethylenebutenolidase [E 3.1.1.45] | Aminobenzoate degradation, Benzoate degradation, Caprolactam degradation | | |

**S5 Table.** The enzyme and xenobiotic degradation and metabolism pathways involved in major genera in the first aerobic bioreactor.
